# EpCAM (CD326) Regulates Intestinal Epithelial Integrity and Stem Cells via Rho-Associated Kinase

**DOI:** 10.3390/cells10020256

**Published:** 2021-01-28

**Authors:** Takeshi Ouchi, Sohshi Morimura, Lukas E. Dow, Hiroyuki Miyoshi, Mark C. Udey

**Affiliations:** 1 Department of Dermatology, Keio University School of Medicine, Shinjuku-ku, Tokyo 160-0016, Japan; takeshi-ouchi.z5@keio.jp; 2 Department of Dermatology, Faculty of Medicine, International University of Health and Welfare, Narita-shi, Chiba 286-8520, Japan; 3 Department of Medicine, Weill Cornell Medicine, New York, NY 10021, USA; lud2005@med.cornell.edu; 4 Institute for Advancement of Clinical and Translational Science (iACT), Kyoto University Hospital, Sakyo-ku, Kyoto 606-8507, Japan; hmiyoshi@mfour.med.kyoto-u.ac.jp; 5 Dermatology Division, Department of Medicine, Washington University School of Medicine, Saint Louis, MO 63110, USA; udey@wustl.edu

**Keywords:** EpCAM, organoid, spheroid, stem cell, ROCK

## Abstract

Humans with biallelic inactivating mutations in Epithelial Cell Adhesion Molecule (EpCAM) develop congenital tufting enteropathy (CTE). To gain mechanistic insights regarding EpCAM function in this disorder, we prepared intestinal epithelial cell (IEC) organoids and spheroids. IEC organoids and spheroids were generated from *ROSA-Cre^ERT2^ EpCAM^fl/fl^* mice. Proliferation, tight junctions, cell polarity and epithelial integrity were assessed in tamoxifen-induced EpCAM-deficient organoids via confocal immunofluorescence microscopy and Western blotting. Olfm4-expressing stem cells were assessed in IEC cells in vitro and in vivo via fluorescence in situ hybridization. To determine if existing drugs could ameliorate effects of EpCAM deficiency in IEC cells, a variety of pharmacologic inhibitors were screened. Deletion of EpCAM resulted in increased apoptosis and attenuated growth of organoids and spheroids. Selected claudins were destabilized and epithelial integrity was severely compromised. Epithelial integrity was improved by treatment with Rho-associated coiled-coil kinase (ROCK) inhibitors without restoration of claudin expression. Correspondingly, enhanced phosphorylation of myosin light chain, a serine/threonine ROCK substrate, was observed in EpCAM-deficient organoids. Strikingly, frequencies of Olfm4-expressing stem cells in EpCAM-deficient IEC cells in vitro and in vivo were decreased. Treatment with ROCK inhibitors increased numbers of stem cells in EpCAM-deficient organoids and spheroids. Thus, EpCAM regulates intestinal epithelial homeostasis via a signaling pathway that includes ROCK.

## 1. Introduction

EpCAM (Epithelial Cell Adhesion Molecule; CD326) is a surface glycoprotein expressed in developing and adult epithelia, and selected carcinomas. EpCAM was initially reported to mediate homophilic adhesion but subsequent studies have suggested that EpCAM is not a typical intercellular adhesion molecule [1]. EpCAM is also expressed by tissue and embryonic stem cells and EpCAM expression is required for stem cell survival or proliferation [2,3]. EpCAM modulates cell motility and migration and it regulates cell mixing at epithelial–dermal interfaces [4]. EpCAM also modulates tight junction composition and function [5]. EpCAM and TROP2 are homologous cell surface proteins and exhibit functional redundancy, but they are not equivalent [6]. In humans and mice, the most prominent feature of mutations in *EpCAM* is development of congenital tufting enteropathy (CTE) [7,8,9,10,11]. CTE is a severe diarrheal syndrome that presents shortly after birth and features severe epithelial dysplasia [7,8].

In mechanistic studies, EpCAM has been reported to be cleaved via regulated intramembrane proteolysis, liberating an intercellular fragment that binds to TCF family transcription factors and modulates expression of several proteins, including c-Myc [12]. EpCAM has also been reported to enable Wnt signaling by inhibiting Kremen1-Dickkopf2-dependent loss of the Wnt co-receptor Lrp6 from cell surfaces [13]. The carboxyl-terminus of EpCAM is homologous to the pseudosubstrate domain of enzymes in the protein kinase C (PKC) family, and loss of EpCAM reportedly activates atypical PKC and distorts actomyosin cytoskeleton remodeling [14]. Several laboratories have reported that EpCAM binds to claudin-7 and claudin-1, preventing these proteins from lysosomal degradation [5,15,16]. Recently, we showed that EpCAM is a matriptase substrate, and that cleavage of EpCAM by matriptase led to internalization and degradation of EpCAM and associated claudins [17]. These results are consistent with the observation that mutations in *SPINT2*, a gene that encodes the matriptase inhibitor HAI-2, also cause CTE [17].

The proposed mechanisms of action of EpCAM may not be convergent or even interrelated. The possibility that EpCAM influences different cellular processes in different cells via distinct mechanisms is also not particularly attractive. Several studies suggest a link between loss of EpCAM and the actomyosin cytoskeleton dysfunction that results in intercellular adhesion and migration anomalies in transformed intestinal epithelial cells in vitro [14,18] and epidermal cells in vivo [19]. Perturbations of the cytoskeleton that result in abnormalities in cell size, shape or stiffness could have severe and pleotropic effects, so perhaps this represents a final common pathway.

Herein, we utilized *ROSA-Cre^ERT^ EpCAM^fl/fl^* transgenic mice that were generated in our laboratory [20] to elucidate key aspects of EpCAM function in several relevant in vitro models. The most prominent feature of mutations in *EpCAM* is CTE. These observations indicate that EpCAM has a non-redundant function in the small intestine and that loss of EpCAM in this tissue leads to a dramatic phenotype. Clevers and coworkers identified conditions that allow propagation and manipulation of primary intestinal epithelial cell (IEC) growing in vitro as organoids that recapitulate key aspects much of IEC growth and differentiation in vivo [21,22,23,24]. Stappenbeck and Miyoshi developed complementary methodology that facilitates the in vitro growth of spheroids of cells with features of intestinal stem cells [25]. We assessed the impact of conditional silencing of EpCAM expression in IEC organoids and spheroids.

We report that EpCAM is essential for maintaining intestinal epithelial homeostasis and intestinal stem cells in mice. Conditional deletion of EpCAM in organoids recapitulated many features of EpCAM loss in vivo and results obtained with IEC organoids led us to hypothesize that EpCAM loss compromised intestinal epithelial stem cell function. Propagation of EpCAM-expressing and EpCAM-deficient stem cell-enriched IEC spheroids confirmed the importance of EpCAM in IEC stem cell function and localized the requirement for EpCAM to stem cells themselves. A systematic search for pharmacologic inhibitors that could blunt the requirement for EpCAM expression revealed that Rho-associated coiled-coil kinase (ROCK) inhibitors and the myosin II inhibitor blebbistatin selectively attenuated the hyperactivation of ROCK that occurs in the absence of EpCAM and improved epithelial integrity and IEC stem cell survival and/or proliferation. We conclude that EpCAM regulates the actomyosin cytoskeleton via a ROCK-dependent mechanism that is critical for optimal function of stem cells and differentiated cells as well.

## 2. Materials and Methods

Please refer to the Supplementary Materials for detailed Materials and Methods.

### 2.1. Mice and Genotyping

B6.129-*Gt(ROSA)26Sor^tm1(cre/ERT2)Tyj^*/J mice were purchased from the Jackson Laboratory (Bar Harbor, ME, USA) and *EpCAM^fl/fl^* mice were generated in our laboratory [20]. Adult (8–12 week old) mice were used in experiments.

### 2.2. IEC Organoid Generation and Propagation

IEC organoids were generated as described [21,22,23,24]. Organoid-forming efficiencies were determined by dividing the number of mature organoids per well on Day 9–11 by the mean number of organoids per well present on Day 1 and multiplying by 100. Organoid passage efficiencies represent the ratio of the absolute number of mature organoids in 5 wells on Day 9 after subculturing (splitting 1 well into 5 wells), and the average number of mature organoids in individual wells on Day 9 before subculturing times 100.

### 2.3. IEC Spheroid Generation and Propagation

IEC spheroids were generated as described [25]. Spheroid-forming numbers were determined by counting numbers of spheroids per well on Day 3 of the 6th passage in the presence and absence of Y27632 (10 μM), H1152 (0.31 μM) and 4-hydroxy tamoxifen (4-OHT) (Sigma-Aldrich, St. Louis, MO, USA, 1 μM). Spheroid passage numbers were determined by counting numbers of spheroids per well on Day 3 of the 7th passage in the continued presence of inhibitors. Spheroids with diameters ≥100 μM were counted.

### 2.4. Deletion of EpCAM In Vitro and In Vivo

To activate *ROSA26 Cre^ERT2^*, isolated crypts or spheroids were incubated with culture medium containing 1 μM of 4-OHT for 72 h. To induce acute *EpCAM* deletion in vivo, adult *ROSA26 Cre^ERT2+^ EpCAM^fl/fl^* mice were treated with tamoxifen (0.2 mg/g body weight, Sigma-Aldrich) daily for 3 days via gavage.

### 2.5. Routine and Confocal Immunofluorescence Microscopy

Organoids and spheroids were fixed in situ with 4% paraformaldehyde in PBS prior to isolation from Matrigel discs. Fixed organoids and spheroids were permeabilized with 0.1% Triton X-100 followed by blocking with 1% bovine serum albumin before overnight incubation with primary antibodies at 4 °C. Phase contrast images of organoids and spheroids were obtained with an inverted microscope (Nikon Eclipse TE2000, Nikon, Minato-ku, Tokyo, Japan). Confocal immunofluorescence images were captured with a laser-scanning confocal microscope (LSM710 or LSM780; Carl Zeiss Micro imaging, Zeiss, White Plains, NY, USA), and data were processed using an LSM Image Browser (Zeiss) and ZEN (Zeiss).

### 2.6. RNA Isolation and qPCR

Wells were rinsed with ice-cold PBS and organoids were harvested by gentle trituration. Total RNA was isolated using Trizol (Invitrogen, Carlsbad, CA, USA) and RNAeasy mini kits (Qiagen, Germantown, MD, USA). cDNA was prepared using AffinityScript Multiple Temperature cDNA Synthesis kits. qPCR was performed using a Bio-Rad CFX96 real-time thermos system (Bio-Rad, Hercules, CA, USA) and PowerSYBR Green PCR master mix (PE Biosystems, Foster City, CA, USA) with Bio-Rad provided PrimerPCR SYBR Green Assay primers. EpCAM mRNA levels are expressed relative to β-actin mRNA as calculated by the 2−ΔΔCT method where ΔCT = CT(EpCAM)−CT(β-actin).

### 2.7. Western Blotting

Wells were rinsed with ice-cold PBS, organoids and spheroids were harvested by pipetting and suspended in lysis buffer. For in vivo experiments, small intestine was homogenized directly into lysis buffer. Equal amounts of protein were incubated in Nupage LDS sample buffer (Invitrogen) and Nupage sample reducing reagent, and resolved in Nupage gels prior to transfer onto polyvinylidene difluoride membranes using an iBlot dry blotting system (Invitrogen). Membranes were blocked with 2% BSA in Tris Buffered Saline with 0.1% Tween20 and then incubated with primary Ab. After incubation with secondary Abs, membranes were exposed to Chemiluminescent Substrate (Thermo Scientific, Caarlsbad, CA, USA, #34075 or #34077) and labeled proteins were detected with photographic film.

### 2.8. EdU Incorporation (Cell Proliferation) Assay

Proliferating IEC in organoids were visualized using a Click-iT EdU imaging kit (Invitrogen) and immunofluorescence microscopy.

### 2.9. TUNEL (Apoptosis) Assay

Apoptotic cells were visualized in IEC organoids using EdUTP and a Click-iT TUNEL imaging kit (Invitrogen) in conjunction with immunofluorescence microscopy.

### 2.10. Transmission Electron Microscopy

Organoids and spheroids in Matrigel were fixed with sodium cacodylate buffer (0.1 M, pH 7.4) containing 2% (*v*/*v*) EM-grade glutaraldehyde (Tousimis, Rockville, MD, USA) and 4% (*v*/*v*) formaldehyde. After processing, grids were examined in an electron microscope (H7600, Hitachi, Dallas, TX, USA) operated at 80 kV. Digital images were captured with a CCD camera (AMT).

### 2.11. Small Molecule Penetration Assay

To assess epithelial integrity, 1 mg/mL EZ-link sulfo-NHS-LC-biotin (ThermoFisher Scientific) was added to organoid culture medium. Organoids were incubated for 4 h at 37 °C, and fixed and permeabilized as described above.

### 2.12. RNA Fluorescence In Situ Hybridization (FISH)

Organoids were cultured for 9 d in the presence and absence of 4-OHT and Y27632 and liberated from Matrigel by pipetting with EDTA/PBS. Pelleted organoids in thrombin/fibrinogen clots were fixed in 4% paraformaldehyde prior to sectioning and hybridization with Defa1, Olfm4, or Defa-1/Olfm4 duplex probes. Slides were counterstained with DAPI and labelled cells were visualized via fluorescence microscopy.

### 2.13. Statistical Analysis

Statistical analysis was performed using the Mann–Whitney *U* test or one-way ANOVA test via Prism5 and 7 software (GraphPad). *p*-values ≤ 0.05 were considered to be significant.

## 3. Results

### 3.1. Murine IEC Organoids Express EpCAM and EpCAM Was Efficiently Deleted in Conditional Knockout (KO) Organoids

**Figure 1:**
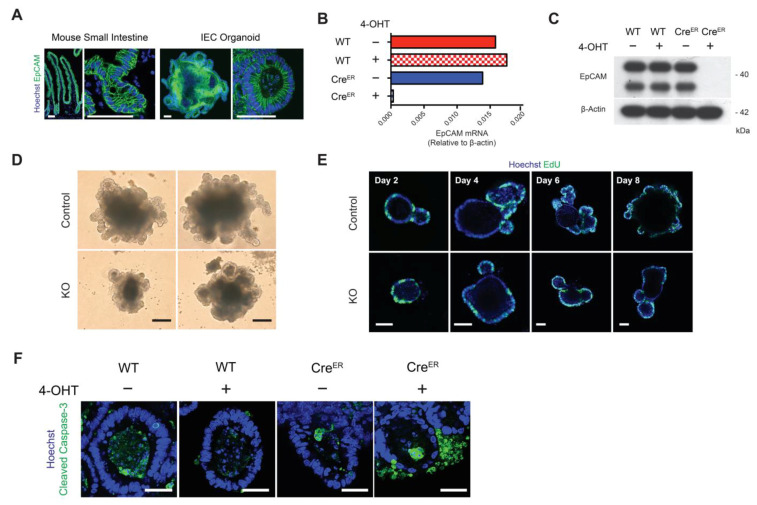
Generation and characterization of Epithelial Cell Adhesion Molecule (EpCAM)-deficient organoids. (**A**) Immunofluorescence microscopy confirming EpCAM expression in mouse small intestine in vivo and intestinal organoids in vitro. EpCAM (green). Bars = 50 μM. (**B**) qPCR demonstrating that EpCAM mRNA is efficiently deleted in KO organoids (normalized to β-actin). (**C**) Silencing of EpCAM protein expression in KO organoids demonstrated by Western blotting. (**D**) Phase contrast photomicrographs of control (untreated *ROSA26-Cre^ERT2+^ EpCAM^fl/fl^*) and KO (4-OHT-treated *ROSA26-Cre^ERT2+^ EpCAM^fl/fl^*) organoids. Bars = 100 μM. (**E**) Assessment of cell proliferation in control and KO organoids after EdU incorporation and fluorescence microscopic detection using Click-iT methodology. Alexa Fluor 488-conjugated EdU (green). Bars = 50 μM. (**F**) Apoptosis in control and KO organoids. Cleaved Caspase-3 (green) detected via immunofluorescence microscopy. Bars = 25 μM. Nuclear counterstaining, Hoechst 33342 (blue) in (**A**,**E**,**F**).

Crypts were isolated and IEC organoids were generated from adult *EpCAM^fl/fl^* and *ROSA-Cre^ERT2^ EpCAM^fl/fl^* mice as previously described [21,22,23,24]. Confocal immunofluorescence microscopy demonstrated that EpCAM was present on all IECs in organoids and that it accumulated at lateral intercellular interfaces (Figure 1A), as is seen in murine small intestine in vivo (Figure 1A).

*EpCAM* was interrupted in developing *ROSA-Cre^ERT2^ EpCAM^fl/fl^* organoids by adding 4-OHT to the culture medium. Exploratory studies revealed that exposure of organoids to 1 μM 4-OHT for the first 3 d of the culture period was required for *EpCAM* silencing (Figure S1A). 4-OHT-induced inhibition of EpCAM expression at the mRNA and protein levels in 8 d organoids was confirmed using qPCR (Figure 1B), Western blotting (Figure 1C) and confocal immunofluorescence microscopy (Figure S1B).

### 3.2. Silencing of EpCAM Led to Smaller Organoids Featuring “Normal” Proliferation but Increased Apoptosis

EpCAM KO organoids were smaller than controls beginning by Day 2 of culture and they were surrounded by subcellular debris (Figure 1D and Figure S1C). Size differences between control and KO organoids increased over the 10 d culture period (Figure S1C). 4-OHT had no effect on organoids from *EpCAM^fl/fl^* mice and untreated *ROSA-Cre^ERT2^ EpCAM^fl/fl^* organoids were indistinguishable from *EpCAM^fl/fl^* organoids (Figure S1C).

Proliferation was assessed using an EdU incorporation assay. Similar frequencies of labeled cells were detected in the budding domains of control and KO organoids at various times during the primary culture period (Figure 1E). Similar levels of p-Histone H3 (Ser 10), a proliferation biomarker, were also detected in KO organoids and all relevant controls (Figure S1D). However, increased frequencies of cleaved caspase-3-expressing cells were detected in KO organoids using confocal immunofluorescence microscopy (Figure 1F) and cleaved caspase-3 was increased in Western blotting of KO organoid lysates (Figure S1E). Cleaved caspase-3- and TUNEL-positive cells in KO cultures (Figure S1F) were located outside organoids rather than in organoid lumens as is the case in control organoids.

### 3.3. IEC Differentiation Occured in EpCAM KO Organoids and Cell Polarity Was Preserved

**Figure 2:**
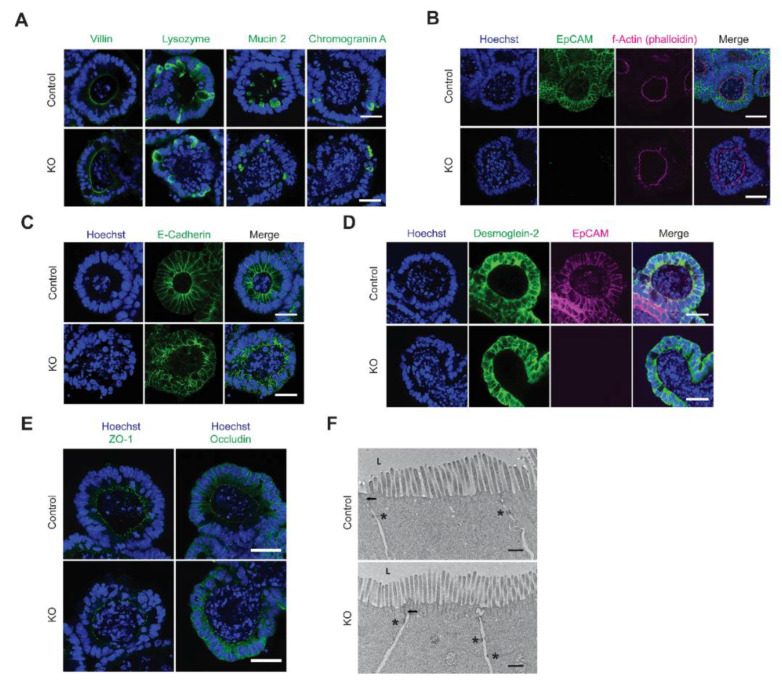
Differentiation, cell polarity and intercellular junctions in EpCAM-deficient organoids. (**A**) Differentiated intestinal epithelial cell (IEC) expressing villin (enterocytes), lysozyme (Paneth cells), mucin 2 (goblet cells) and chromogranin A (enteroendocrine cells) developed in control and KO organoids. Specific staining (green). Bars = 25 μM. (**B**) Localization of actomyosin cytoskeleton in control and KO organoids. EpCAM (green); phalloidin (pink). Bars = 25 μM. (**C**) Detection and localization of E-cadherin in control and KO organoids. E-Cadherin (green). Bars = 25 μM. (**D**) Expression and localization of desmoglein-2 and EpCAM in control and KO organoids. Dsg-2 (green); EpCAM (pink). Bars = 25 μM. (**E**) Expression and localization of ZO-1 and occludin in control and KO organoids. Specific staining (green). Bars = 25 μM. (**F**) Transmission electron micrographs of control and KO organoids. Arrows indicate tight junctions and asterisks designate desmosomes (L = bud lumens). Bars = 500 n. Nuclear counterstaining, Hoechst 33342 (blue) in (**A**–**E**).

IEC organoids support the development of all IEC lineages from stem cells in vitro. Immunofluorescence microscopy demonstrated that lysozyme-expressing Paneth cells, mucin 2-expressing goblet cells and chromogranin A-expressing enteroendocrine cells were all represented in control and KO organoids (Figure 2A). Cell polarity was also preserved in KO organoids. This was manifested by subapical localization of the F-actin-containing cytoskeleton in control and KO organoids (Figure 2B) and the presence of Na^+^/K^+^ ATPase on lateral and basal IEC surfaces (Figure S2A).

### 3.4. Intercellular Junctional Complexes Were Present in Disordered Epithelium in EpCAM KO Organoids

Using confocal immunofluorescence microscopy (Figure 2C–E) and Western blotting (Figure S2B,C), we determined that components of adherence junctions (E-cadherin), desmosomes (desmoglein-2) and tight junctions (ZO-1 and occludin) were detected, were similarly distributed and were present at similar levels in control and KO organoids. Staining with anti-E-cadherin Ab revealed that the arrangement of IEC in KO organoid budding domains was disordered, however (Figure 2C). Tight junctions and desmosomes were readily identified in transmission electron photomicrographs of samples from control and KO organoids (Figure 2F). Close inspection revealed that ~30% of KO desmosomes were asymmetric (did not have two dense plaques), while only ~5% of control desmosomes were asymmetric (Figure S2D,E), and that gaps between desmosome faces in KO organoids were ~2× that in control organoids.

### 3.5. Selected Claudins Were Destabilized in KO Organoids and Epithelial Integrity Was Compromised

**Figure 3:**
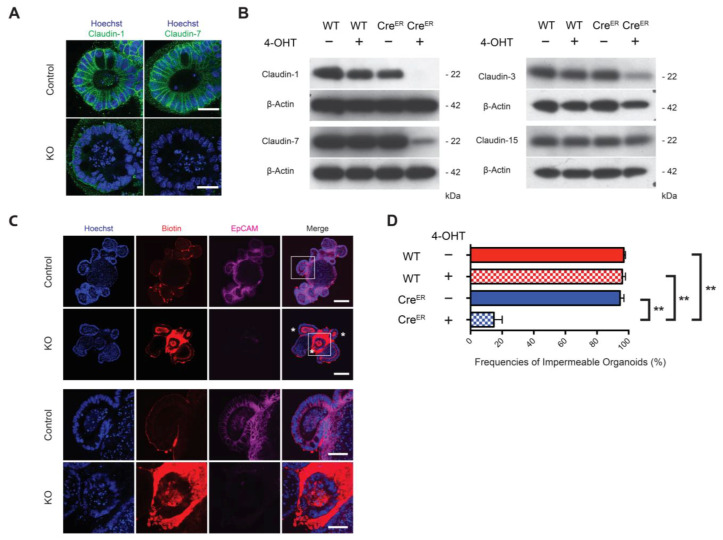
Reduced claudins and compromised epithelial integrity in EpCAM-deficient organoids. (**A**) Reductions in claudin-1 (left) and claudin-7 (right) expression in KO organoids. claudin staining (green). Bars = 25 μM. (**B**) Reduction of claudins in KO organoids by Western blotting. WT represents *EpCAM^fl/fl^* and Cre^ER^ represents *ROSA26-Cre^ERT2+^ EpCAM^fl/fl^* orgnoids. Results from 1 of 3 independent experiments. (**C**) Organoid permeability (epithelial integrity) was assessed via fluorescence microscopy after sulfo-NHS-LC-biotin (red) treatment. Bars = 100 μM. Expanded views (lower panels) reveal labeling of KO organoids. Bars = 25 μM. (**D**) Quantitation of organoid permeability. In each experiment in each condition, 50 organoids were randomly selected and frequencies of organoids with labeled luminal contents were determined. Data depicted represent aggregate data from three independent experiments (mean ± SEM; ** *p* < 0.01 via Student’s *t* test). Nuclear counterstaining, Hoechst 33342 (blue) in (**A**,**C**).

We assessed levels of expression of selected claudins in EpCAM-expressing and KO organoids using confocal immunofluorescence microscopy (Figure 3A) and Western blotting (Figure 3B). While ZO-1 and occludin expression was preserved, loss of EpCAM was associated with the downregulation of claudin-7 and claudin-1 as well as claudin-3. Claudin-15 expression was not compromised.

We assessed the permeability of control and KO organoids to a small molecule as an alternative indicator of epithelial function. Sulfo-NHS-LC-biotin (MW 556.59 Da) was added into the media surrounding organoids and the incubation was continued for 4 h (Figure 3C). Luminal contents of KO organoids were frequently labeled while labeling was restricted to the periphery of control organoids. Whereas ~95% of all control organoids excluded sulfo-NHS-LC-biotin, only ~15% of KO organoids were sulfo-NHS-LC-biotin impermeable (Figure 3D). 

### 3.6. Epithelial Integrity in KO Organoids Was Selectively Improved by Treatment with ROCK Inhibitors

**Figure 4:**
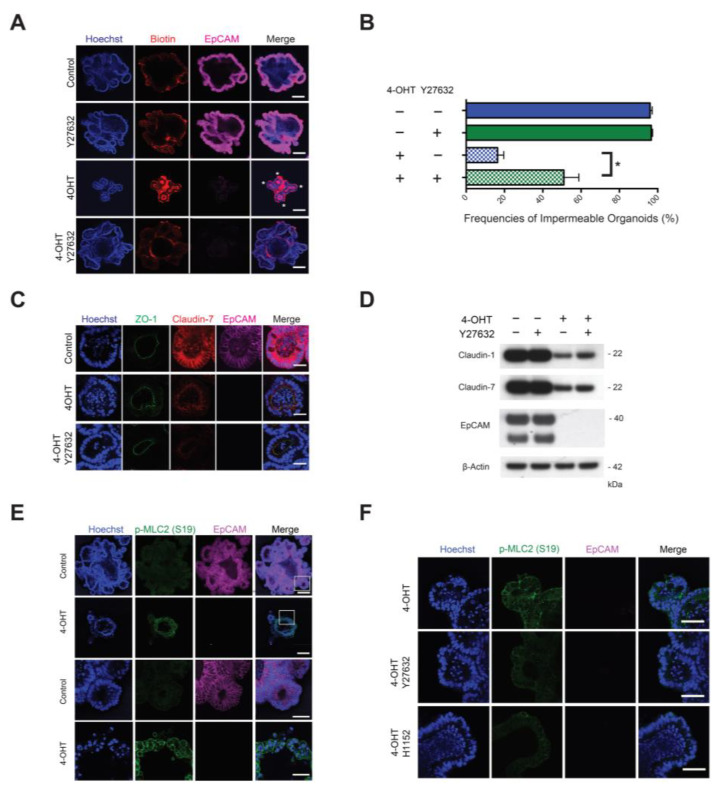
Rho-associated coiled-coil kinase (ROCK) inhibitors attenuate myosin light chain 2 (MLC2) phosphorylation and improve epithelial integrity in EpCAM KO organoids without modulating claudin levels. (**A**) KO organoid permeability to sulfo-NHS-LC-biotin (red) was assessed with, and without, Y27632 treatment. Asterisks highlight labeled budding lumens with compromised epithelial integrity. Bars = 100 μM. (**B**) Quantitation of Y27632-induced improvement of epithelial integrity. Permeability of organoids to sulfo-NHS-LC-biotin was assessed by confocal fluorescence microscopy. In each condition in each experiment, 50 randomly selected organoids were scored. Data depicted represent aggregate data from three independent experiments (mean ± SEM; * *p* < 0.05 via Student’s *t* test). (**C**) Assessment of claudin-7 expression (red) in KO organoids with, and without, Y27632 treatment. Bars = 25 μM. (**D**) ROCK inhibitor treatment of EpCAM-deficient organoids does not restore EpCAM or claudins expression. Organoid lysates were immunoblotted with anti-claudin-1, claudin-7, EpCAM and β-actin Ab. Representative data from 1 of 3 independent experiments are shown. (**E**) Phosphorylation of MLC2 (S19) (green) in KO organoids on Day 9. Bars = 100 μM. Lower panels represent expanded views of regions designated in the upper panel. Bars = 25 μM. (**F**) Inhibition of phosphorylation of MLC2 (S19) (green) in KO organoids by treatment with the ROCK inhibitors Y27632 and H1152 on Day 9. Bars = 25 μM. EpCAM (pink), Hoechst 33342 (blue) in (**A**,**C**,**E**,**F**). Representative data from 1 of 3 independent experiments are show in (**A**,**C**–**F**).

We screened a variety of pharmacologic inhibitors to identify pathways that might be regulated by EpCAM and might influence epithelial integrity. In the initial screen, only the ROCK inhibitor Y27632 improved epithelial integrity (Figure 4A,B). Development of organoids with restored epithelial integrity did not reflect outgrowth of stem cells that expressed EpCAM (Figure 4A,C,D), and Y27632 did not result in re-expression of claudin-7 or claudin-1 (Figure 4C,D).

We predicted that ROCK I and/or ROCK II activity should be increased in EpCAM KO organoids. We failed to document increased ROCK activity or increased accumulation of phosphorylated ROCK substrates in lysates of KO organoids using conventional biochemical assays (direct measurement of enzyme activity and Western blotting of phosphorylated proteins). When we assessed phosphorylation of ROCK substrates in situ using Ab reactive with phosphorylated myosin light chain 2 (p-MLC2) and confocal immunofluorescence microscopy and we found that staining with anti-p-MLC2 (S19) and anti-p-MLC2 (T18/S19) Ab was increased in KO organoids (Figure 4E and Figure S3A). Treatment of KO organoids with Y27632 and H1152, another ROCK inhibitor, reduced reactivity with anti-p-MLC2(S19) and anti-p-MLC2(T18/S19) (Figure 4F and Figure S3B). Mean intensity of p-MLC2(T18/S19) staining in EpCAM KO organoids was significantly higher than that of control organoids and staining was attenuated by the ROCK inhibitors (Figure S3A–C). These results indicate that downstream of the ROCK pathway is increased in EpCAM KO organoids and suggest that Y27632 and H1152 modify epithelial integrity by inhibiting the ROCK pathway.

### 3.7. IEC Stem Cells Were Compromised in EpCAM KO Organoids/Spheroids and EpCAM KO Mice

**Figure 5:**
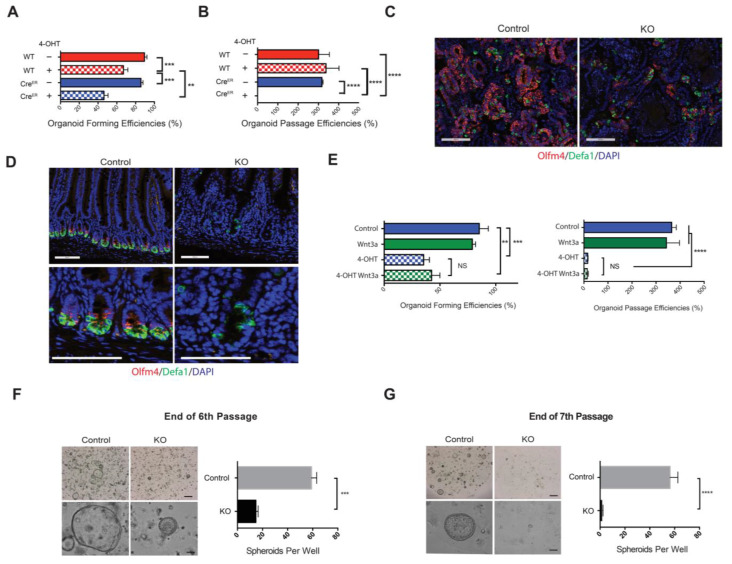
Effects of EpCAM loss on IEC stem cells and/or stem cell function. (**A**) KO organoids form less efficiently than control organoids. Efficiencies represent ratios of organoid numbers/well at Day 9 over crypt numbers/well on Day 1. Aggregate data from 3 independent experiments are depicted. ** *p* < 0.01 and *** *p* < 0.001 via Student’s *t* test. (**B**) KO organoids cannot be subcultured. Organoid passage efficiencies represent ratios of organoid numbers/well at Day 9 of secondary cultures over organoid numbers/well at Day 9 of primary cultures. Representative results from 1 of 2 independent experiments are shown. **** *p* < 0.0001 via Student’s *t* test. (**C**) Abnormalities in stem cells and Paneth cells in KO organoids at Day 9 revealed via FISH (Olfm4 (red); Defa1 (green); DAPI (blue)). Bars = 100 μM. Data from 1 of 3 independent experiment are shown. (**D**) Small intestinal epithelial stem cells and Paneth cells in EpCAM-deficient mice on Day 7 after initiation of tamoxifen treatment identified by FISH (Olfm4 (red); Defa1 (green); DAPI (blue)). Bars = 100 μM. Data from 1 of 3 independent experiments are shown. (**E**) Forming and passaging efficiencies of KO organoids with, and without, treatment with exogenous Wnt-3a (100 ng/mL). ** *p* < 0.01, *** *p* < 0.001 and **** *p* < 0.0001 via Student’s *t* test. NS; not significant. Data from 1 of 3 independent experiments are shown. (*F* and *G*) Phase contrast photomicrographs and numbers of control and EpCAM KO spheroids on Day 3 of the 6th passage (**F**) or on Day 3 of the 7th passage (**G**). Bars = 500 μ (upper), 100 μM (lower). *** *p* < 0.001 and **** *p* < 0.0001 via Student’s *t* test. Representative images from 1 of 3 independent experiments and aggregate data from three independent experiments are shown in (**F**,**G**). 4-OHT was added at the beginning of passage 6. Data depicted represent mean ± SEM in (**A**,**B**,**E**–**G**).

Compromised IEC stem cell function could result in reduced organoid-forming or passage efficiencies. Quantification of organoid-forming efficiencies revealed a ~50% reduction after EpCAM depletion in primary cultures (Figure 5A) and EpCAM-deficient organoids could not be passed (Figure 5B). In our experiments, *EpCAM* was deleted over a period of several days. Thus, EpCAM was present in stem cells at the initiation of all cultures but is absent from stem cells at the time of passage. We carried out FISH experiments using Olfm4- and Defa1-specific probes to detect stem cells and Paneth cells, respectively. Frequencies of Olfm4^+^ cells were dramatically reduced in Day 9 KO organoids as compared with controls (Figure 5C). Frequencies of Defa1^+^ Paneth cells were also decreased in KO organoids and their morphology was abnormal (Figure 5C).

Germline and intestine-selective EpCAM KO mice develop CTE within the first few days of life. We treated *ROSA-Cre^ERT2^ EpCAM^fl/fl^* and control mice with tamoxifen (0.2 mg/g body weight in oil on Days 0–2) or vehicle (oil) by gavage and measured body weights serially. *ROSA-Cre^ERT2^ EpCAM^fl/fl^* mice that were treated with tamoxifen lost weight, requiring euthanasia by Day 7 (Figure S4A). At necropsy, EpCAM protein was not present in the Western blotting of intestinal tissue lysates of tamoxifen-treated *ROSA-Cre^ERT2^ EpCAM^fl/fl^* mice (Figure S4B). These mice also selectively exhibited the epithelial tufts and expanded crypts that are typical histologic features of CTE (Figure S4C). Assessment of stem cell and Paneth cell frequencies in the intestines of acute EpCAM KO mice using FISH revealed dramatic decreases in both types of cells, as we had previously observed in IEC KO organoids (Figure 5D).

### 3.8. Intestinal Epithelial Stem Cells Required EpCAM for Survival and/or Proliferation

Generation of IEC organoids requires preservation of crypt architecture that approximates stem cells to Wnt3a-producing Paneth cells [22]. Disruption of this relationship or loss of Paneth cells results in decreased organoid-forming ability. To determine if EpCAM KO organoid dysfunction was related to loss of Paneth cells, we assessed effects of exogenous Wnt3a on EpCAM KO organoid-forming and passaging efficiencies. We also assessed the protein levels of phospho-beta-catenin, which regulates Wnt/beta-catenin signaling, in EpCAM KO organoids by Western blotting and immunofluorescence microscopy. We did not find significant differences between controls and EpCAM KO organoids (data not shown). Thus, we have no evidence that EpCAM regulates stem cell homeostasis in our experiments via the Wnt/beta-catenin signaling pathway. The inability of Wnt3a to augment KO organoid formation or passage efficiencies suggests an intrinsic stem cell defect rather than dysfunction secondary to Paneth cell loss or failure (Figure 5E).

Stappenbeck and colleagues have defined conditions that allow for the propagation of stem cell-enriched spheroids from mouse intestine [25]. We utilized this approach to generate spheroids from *ROSA-Cre^ERT2^ EpCAM^fl/fl^* mice (Figure S5A). In the absence of 4-OHT, all cells in spheroids expressed EpCAM while 4-OHT-treated spheroids showed markedly reduced EpCAM expression (Figure S5B,C). In subsequent experiments, spheroids were subsequently treated with 4-OHT (1 μM) during the 6th passage as indicated in the schematic Figure S5D, and spheroid-forming and passage numbers were measured. The number of KO spheroids present at the end of the 6th passage was ~25% of that present in control spheroid cultures, and the KO spheroids that were present were small (Figure 5F). Extremely few KO spheroids were present at the end of Passage 7 (Figure 5G). We confirmed that differentiated cells, including Paneth cells, were eliminated from spheroid cultures by serial passage (Supplementary Figure S5E–G), indicating that studies of spheroids are a reasonable surrogate for studies of IEC stem cells.

Results obtained with EpCAM-deficient spheroids were consistent with in vivo studies. *ROSA-Cre^ERT2^ EpCAM^fl/fl^* mice were treated with tamoxifen or vehicle and the ability of Day 7 crypts to generate IEC organoids and spheroids was tested. EpCAM KO intestinal epithelium did not support formation of IEC organoids or spheroids (Figure S6A–D). These results suggest that IEC stem cells must express EpCAM to survive and proliferate both in vitro and in vivo.

### 3.9. ROCK Inhibitors Mitigated Effects of EpCAM Loss in IEC Stem Cells

**Figure 6:**
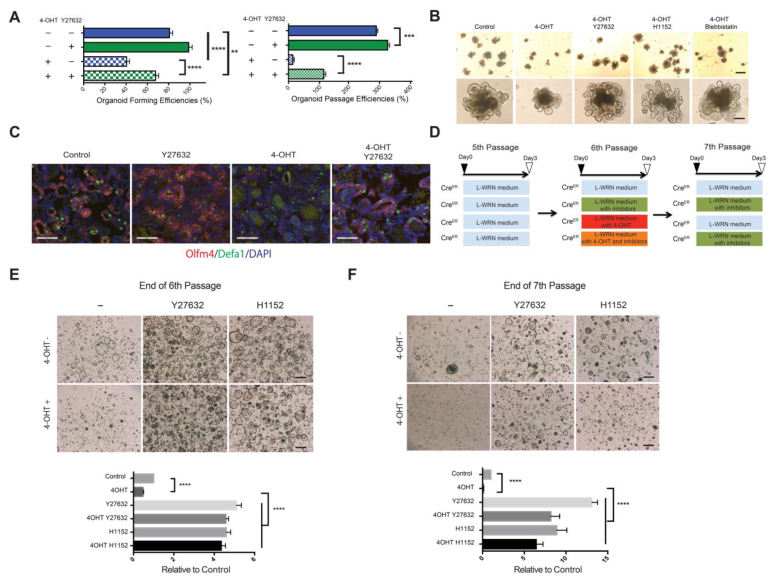
ROCK and myosin II inhibitors attenuate abnormalities in EpCAM KO IEC organoids and spheroids. (**A**) Y27632 significantly improved organoid-forming and passaging efficiencies in KO organoids. Results from 1 of 3 independent experiments (organoid forming) and 1 of 2 independent experiments (organoid passaging) are depicted. ** *p* < 0.01, *** *p* < 0.001 and **** *p* < 0.0001 via Student’s *t* test. (**B**) Phase contrast photomicrographs of IEC organoids with and without treatment with 4-OHT (for the initial 3 d) and pharmacologic inhibitors (for the entire 9 d) of the culture period as indicated. Y27632 (10 μM), H1152 (0.31 μM) and blebbistatin (10 μM) were tested. Bars = 250 μ (upper panels), 100 μ (lower panels). (**C**) Detection of stem cells ((Olfm4^+^, red); Paneth cells (Defa1^+^, green)) using FISH and control or EpCAM KO organoids in the presence and absence of Y27632 at Day 9 as indicated. DAPI (blue). Bars = 100 μM. Data from 1 of 2 independent experiments are shown. (**D**) Strategy for drug treatment and passaging of IEC spheroids. Sixth passage spheroids were treated with 4-OHT in the presence and absence of Y27632 or H1152 for 3 d as indicated. ROCK inhibitors were also added to the media used to propagate 7th passage spheroids as indicated. (**E**,**F**) Phase contrast photomicrographs and numbers of control and EpCAM KO ROCK inhibitor-treated spheroids on Day 3 of the 6th passage (**E**) or Day 3 of the 7th passage (**F**). Bars = 500 μM. **** *p* < 0.0001 via Student’s *t* test. Data depicted represent mean ± SEM in (**A**,**E**,**F**). Images from 1 of 2 independent experiments and aggregate data from two independent experiments are shown in (**E**,**F**).

Because ROCK inhibitors restored epithelial integrity in EpCAM KO organoids, we hypothesized that ROCK inhibitors would augment EpCAM KO stem cell function. Addition of Y27632 (10 μM) into cultures increased KO organoid efficiency by ~2-fold and KO organoid passage efficiency by >5-fold (Figure 6A). Y27632 also progressively increased the number of budding domains of EpCAM KO organoids (Figure 6B and Figure S7), Another ROCK inhibitor (H1152) and the myosin II inhibitor blebbistatin had analogous effects (Figure 6B) confirming the involvement of ROCK in EpCAM-dependent stem cell dysfunction and implicating EpCAM as a modulator of cytoskeletal remodeling.

To determine if Y27632 increased stem cell survival or proliferation, we enumerated stem cells and Paneth cells in control and EpCAM-deficient organoids in the presence and absence of Y27632 using FISH. Y27632 treatment increased frequencies of Olfm4^+^ stem cells in control organoids and dramatically increased numbers of Olfm4^+^ stem cells and Defa1^+^ Paneth cells in KO organoids (Figure 6C).

We studied IEC spheroids to determine if ROCK inhibitors were acting directly on stem cells using the strategy outlined in Figure 6D. *ROSA-Cre^ERT2^ EpCAM^fl/fl^* 6th passage spheroids were treated with 4-OHT and ROCK inhibitors and then subcultured in ROCK inhibitors in the absence of tamoxifen. Spheroid numbers were determined at the end of the 6th and 7th passages to assess spheroid-forming and passage efficiencies, respectively. As expected, deletion of EpCAM decreased spheroid-forming and passage efficiencies. In the case of control spheroids, Y27632 (10 μM) and H1132 (0.31 μM) increased spheroid-forming efficiencies ~5-fold and passage efficiencies >10-fold (Figure 6E,F). Both inhibitors also blunted effects of EpCAM loss on spheroid formation and passage efficiencies. ROCK inhibitors did not exert their effects by promoting the outgrowth of EpCAM-expressing stem cells (Figure S8). These results implicated EpCAM as a regulator of ROCK in IEC and indicate that EpCAM-dependent regulation of ROCK is critical for IEC stem cell survival and/or proliferation.

To determine if ROCK inhibitors were selectively able to ameliorate effects of EpCAM deficiency in IEC stem cells, we screened a variety of pharmacologic inhibitors using the strategy depicted in Supplementary Figure S9A. The selective ROCK I inhibitors Y27632, H1152 and Fasudil, as well as blebbistatin, improved passage of EpCAM-deficient spheroids while a selective ROCK II inhibitor, KD025, did not (Figure S9B). A previous study reported that EpCAM acts as endogenous inhibitor of nPKC, thereby regulating actomyosin contractility and cell adhesion [14]. To assess the possible involvement of EpCAM/PKC interactions in IEC spheroids, we tested following PCK inhibitors; bisindolylmaleimide-I (a generic PKC inhibitor), calphostin C (a PKCγ,δ inhibitor), Go6976 (a PKCα,β,γ inhibitor), PKC20-28 (a PKCα,β inhibitor), a PKCeta inhibitor and a PKCzeta inhibitor. None of these agents promoted growth of EpCAM KO spheroids at concentrations that were reported in the literature to be active (Figure S9B). Thus, we have no indication that EpCAM regulates PKC in IEC spheroids. Other candidate inhibitors including Rapamycin (an mTOR inhibitor), FIPI (a phospholipase D inhibitor), SP600125 (a JNK inhibitor) and Cytochalasin D (an actin polymerization inhibitor) also did not promote EpCAM KO spheroid propagation (Figure S9B). Inhibitors of ROCK I and blebbistatin appear to be uniquely able to prevent adverse downstream effects of EpCAM loss in IEC stem cells.

## 4. Discussion

In the present study, we utilized relevant in vivo and in vitro models to explore EpCAM function in murine IEC. Tamoxifen treatment of *ROSA-Cre^ERT2^* transgenic *EpCAM^fl/fl^* mice as well as IEC organoids and spheroids from these animals rapidly and efficiently silenced *EpCAM* expression and recapitulated key features of CTE. Selected claudins were destabilized, epithelial integrity was compromised, apoptosis was enhanced and stem cell function was impaired. Although Paneth cells were rapidly lost from EpCAM-deficient mice and organoids, stem cell function was not restored by exogenous Wnt3a and stem cell function was also severely compromised in spheroid cultures that lacked differentiated cells. EpCAM expression is required for IEC stem cell proliferation and/or survival.

Candidate pharmacologic inhibitors were employed to gain mechanistic insights. ROCK I inhibitors and the myosin II inhibitor blebbistatin selectively reversed the effects of EpCAM loss in IEC in vitro without restoring claudin expression, suggesting at least two distinct mechanisms by which EpCAM regulates cellular physiology (one claudin-dependent and the other not). We could not document the association of ROCK I with EpCAM, and the mechanism by which EpCAM regulates ROCK in this experimental system has not been delineated. The ability of blebbistatin treatment to reverse effects of EpCAM loss does implicate the ROCK substrate myosin light chain kinase, phosphorylated myosin light chain, myosin II and the actomyosin cytoskeleton in the functional pathway that includes EpCAM and ROCK, however.

Previous studies of EpCAM in the development of xenopus integument demonstrated that EpCAM functioned as an endogenous inhibitor of atypical PKC and that dysregulation of this enzyme was upstream of ROCK activation and myosin light chain phosphorylation [14]. We were unable to implicate EpCAM loss-associated PKC activation in IEC organoids and/or spheroids using standard biochemical approaches as well as multiple well-characterized small molecule inhibitors and several robust assays (epithelial integrity and stem cell function) in which ROCK inhibitors and blebbistatin had potent activity. This suggests that the mechanism by which EpCAM regulates ROCK in murine IEC may be different from that in xenopus integument.

Several additional publications suggested a relationship between EpCAM expression and regulation of cytoskeletal function. Overexpression of EpCAM in thymic epithelial cells led to reorganization of the actin cytoskeleton with the formation of stress fibers and cellular protrusions [26]. Recently, Salomon and coworkers demonstrated that loss of EpCAM in human IEC led to disorganization of epithelial cell surfaces reflected by brush border abnormalities, tight junction belt irregularities, mislocation of tricellular junctions and cell shape changes [18]. In studies of Caco2 cells, these investigators associated loss of EpCAM with local upregulation of myosin light chain kinase at sites of tricellular junctions in epithelial monolayers. Interestingly, loss of EpCAM in Caco2 cells led to global decreases in p-MLC with selectively enhanced p-MLC accumulation at abnormal tricellular junctions. Their findings indicated local impairment of cytoskeletal remodeling, and were consistent with the observation that the myosin II inhibitor blebbistatin normalized tricellular junction structure, and reversed other abnormalities in EpCAM-deficient cells as well. Tricellulin loss did not phenocopy the effects of EpCAM silencing, however, suggesting that effects of EpCAM loss are not mediated exclusively through tricellular junction perturbations. In organoids, we observed a diffuse pattern of p-MLC accumulation in EpCAM-deficient IEC consistent with global effects on the actomyosin cytoskeleton. We did not identify striking abnormalities in intercellular junctions or in brush borders, but is possible that our studies had less resolving power than those of Salomon et al. Independent of these differences, both studies clearly implicate dysregulated myosin II and perturbation of actomyosin network homeostasis as essential features of EpCAM loss in IEC.

Cell shape, stiffness and motility must be carefully regulated in epithelial cells that comprise a dynamic tissue in which cells must proliferate, differentiate, migrate and die while preserving necessary proximities, adhering to each other and maintaining the barrier between organism and the environment. Thus, it is not difficult to understand how hypercontractility of the actomyosin cytoskeleton in the setting of EpCAM loss could lead to catastrophic failure of the intestinal epithelium as is seen in CTE. It is less obvious that EpCAM loss and enhanced MLC phosphorylation should compromise stem cell function, but this observation is also entirely consistent with previous studies [14,18]. The ability of ROCK inhibitors to promote the survival and proliferation of primary cells, including embryonic and tissue stem cells, is well known [27,28]. Several years ago, multiple groups simultaneously reported that cell dissociation-induced apoptosis of human embryonic stem cells was associated with ROCK activation and myosin light chain phosphorylation, and that this programmed cell death could be prevented by treatment with ROCK inhibitors and blebbistatin, and genetic silencing of myosin II [27,28,29,30]. Related to this, Zhao and coworkers recently reported that the deletion or silencing of non-muscle myosin II in murine IEC protects against experimental colitis and promotes survival of Lgr5+ stem cells and growth of IEC organoids [31]. Salomon and coworkers showed that loss of actomyosin network and contractile activity clustering in CTE were recovered by myosin-II inhibitor treatment, which is consistent with our present data [18]. Expression of high levels of EpCAM is a feature of all IEC and human embryonic stem cells, and we propose that regulation of actomyosin network dynamics by EpCAM is critical for normal stem cell growth and/or survival and intestinal epithelial homeostasis.

We have recently demonstrated that, in IEC, EpCAM expression is regulated via a novel post-transcriptional mechanism. Cleavage of EpCAM by the cell surface protease matriptase leads to the internalization and lysosomal degradation of EpCAM and the physically associated proteins claudin-1 and claudin-7 [17]. In normal cells, humans and mice, matriptase activity is restrained by the simultaneous expression of the cell surface protease inhibitor HAI-2 (encoded by *SPINT2*). Loss or inactivation of *SPINT2* phenocopies loss of EpCAM in vitro and in patients, suggesting that this pathway is relevant to normal physiology. We have also showed that EpCAM and TROP2 exhibit redundancies with regard to the regulation of claudin metabolism and the regulation of an HAI, matriptase, EpCAM and claudin pathway [32]. In future studies, it will be important to assess the extent to which EpCAM expression by stem cells is regulated via matriptase and to determine if actomyosin cytoskeletal dynamics in stem cells can also be regulated via this mechanism.

## Data Availability

Not applicable.

## Supplementary Materials

The following are available online at https://www.mdpi.com/2073-4409/10/2/256/s1, Figure S1: Generation and characterization of EpCAM-deficient organoids. Figure S2: Cell polarity and intercellular junctions in EpCAM-deficient organoids. Figure S3: Attenuation of enhanced MLC2 (T18/S19) phosphorylation in KO organoids by the ROCK inhibitors. Figure S4: Clinical and histological features of tamoxifen-treated *ROSA-Cre^ERT2+^ EpCAM^fl/fl^* mice mimic those of patients with CTE. Figure S5: Establishment and characterization of EpCAM-expressing and EpCAM-deficient IEC spheroids. Figure S6: IEC organoids and spheroids cannot be efficiently generated from mice in which EpCAM has been acutely deleted. Figure S7: Effects of Y27632 on control and KO IEC organoids. Figure S8: KO spheroids that are propagated in ROCK inhibitors do not express EpCAM. Figure S9: Effects of ROCK1 and myosin II inhibitors on EpCAM KO IEC spheroids are selective. Supplementary Materials and Methods.

## Abbreviations

CTE	congenital tufting enteropathy
PKC	protein kinase C
IEC	intestinal epithelial cells
4-OHT	4-hydroxy tamoxifen
ROCK	Rho-associated coiled-coil kinase
KO	knockout
p-MLC2	phosphorylated myosin light chain 2
FISH	fluorescence in situ hybridization
