# Supplementary material for: EpCAM (CD326) Regulates Intestinal Epithelial Integrity and Stem Cells via Rho-Associated Kinase
Source: Cells. 2021 Jan 28;10(2):256. doi: 10.3390/cells10020256 (PMC7912093; doi:10.3390/cells10020256)
Supplement: Supplementary file 1 [file cells-10-00256-s001.pdf]

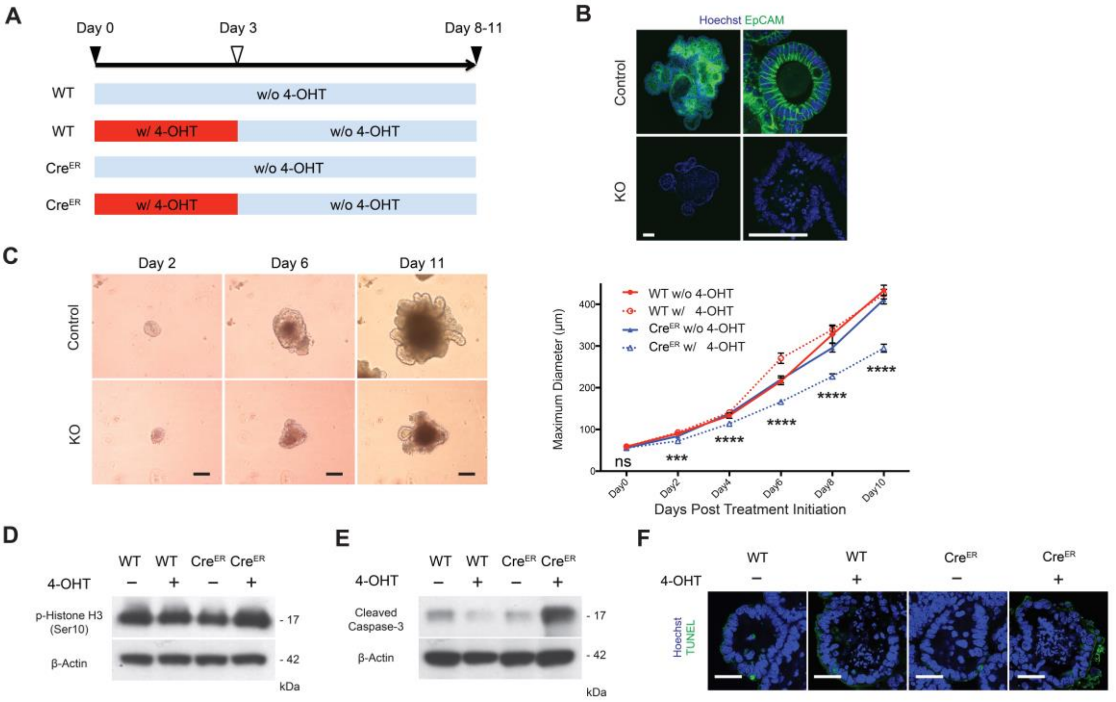

Figure S1. Generation and characterization of EpCAM-deficient organoids. (*A*) Schematic describing strategy for inducible deletion of EpCAM in IEC organoids from *ROSA26-Cre*$^{ERT2+}$ *EpCAM*$^{fl/fl}$ mice *in vitro*. (*B*) Confirmation of conditional deletion of EpCAM in KO organoids demonstrated by confocal immunofluorescence microscopy. EpCAM (green); Hoechst 33342 counterstaining (blue). Bars = 50 µ. Control corresponds to untreated *ROSA26-Cre*$^{ERT2+}$ *EpCAM*$^{fl/fl}$ and KO corresponds to 4-OHT-treated *ROSA26-Cre*$^{ERT2+}$ *EpCAM*$^{fl/fl}$ organoids. (*C*) Time course of growth of EpCAM-expressing and EpCAM-deficient organoids *in vitro*. Left panel, representative phase contrast photomicrographs. Right panel, growth curves depicting mean maximal diameters of 50 randomly selected organoids for each condition at the indicated time points. *** $P < .001$ and **** $P < .0001$ in comparisons between 4-OHT-treated organoids derived from WT and cKO mice using a one-way ANOVA test (mean ± SEM). Bars = 100 µ. (*D* and *E*) Levels of expression of the proliferation biomarker phospho-histone H3 (Ser10) and the apoptosis biomarker cleaved caspase-3 were assessed in IEC organoid lysates by Western blotting. (*F*) Detection of apoptotic cells via TUNEL assay. TUNEL^{+} cells (green), Hoechst 33342 counterstain (blue). Bars = 25 µ. Representative results from 1 of 3 independent experiments are shown.

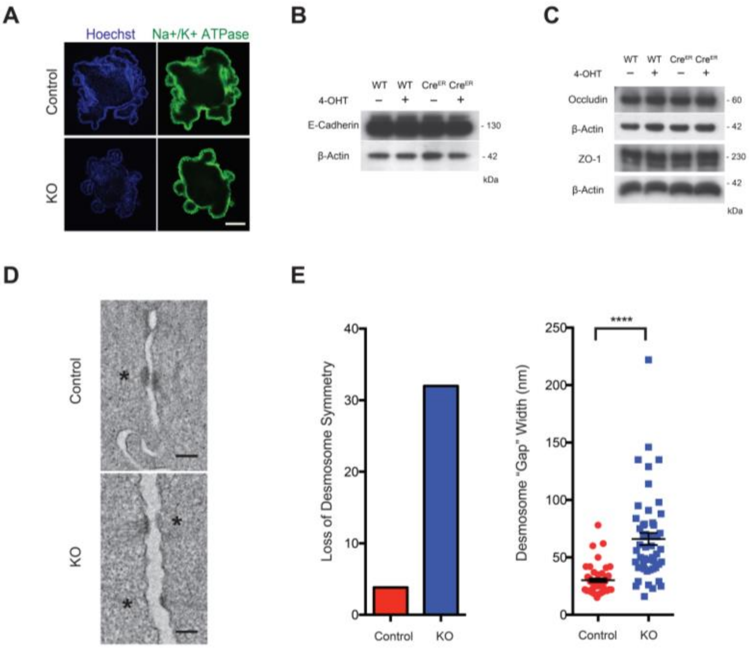

Figure S2. Cell polarity and intercellular junctions in EpCAM-deficient organoids. (*A*) Expression and localization of Na+/K+ ATPase in control and KO organoids determined using confocal immunofluorescence microscopy. Bars = 100 µ. (*B*) E-Cadherin expression in organoids estimated via Western blotting of organoid lysates. Representative results shown are from 1 of 2 independent experiments. (*C*) Assessment of expression of selected tight junction-associated proteins in control and KO organoids by Western blotting of organoid lysates. Representative results depicted are from 1 of 2 independent experiments. (*D*) High magnification of desmosomes of control and KO organoids in transmission electron micrographs. Asterisks designate desmosomes. Bars = 100 n. (*E*) Loss of symmetry and increased inter-plaque distances desmosome in KO organoids (based on analysis of 52 control and 53 KO) randomly selected desmosomes. Each dot represents an individual junction (mean ± SEM). **** $P < .0001$ via Student's t test.

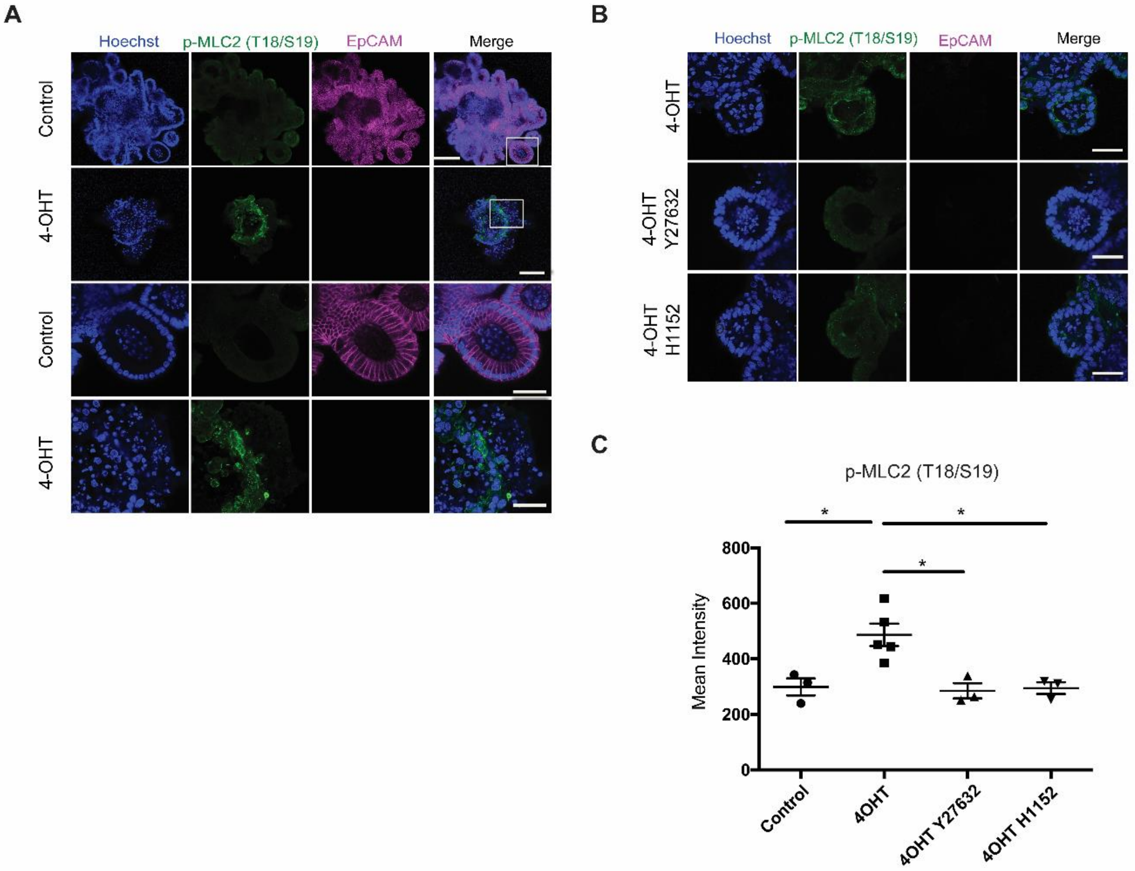

Figure S3. Attenuation of enhanced MLC2 (T18/S19) phosphorylation in KO organoids by the ROCK inhibitors. (*A*) Assessment of phosphorylation of MLC2 (T18/S19) (green) in control and KO organoids on Day 9 via confocal immunofluorescence microscopy. Bars = 100 μ. Lower panels represent expanded views of areas designated in upper panels. Bars = 25 μ. (*B*) Inhibition of enhanced phosphorylation of MLC2 (T18/S19) (green) in KO organoids by Y27632 and H1152 treatment on Day 9 (upper panel). Bars = 25 μ. EpCAM (pink), Hoechst 33342 (blue) in (*A*) and (*B*). (*C*) Quantification of p-MLC2 (T18/S19) immunofluorescence mean pixel intensity among controls, KOs and KOs with ROCK inhibitors Y27632 and H1152. Each dot represents individual samples (mean ± SEM). *$P < .05$ via Student's t test. Representative data from 1 of 3 independent experiments are shown in (*A*) and (*B*).

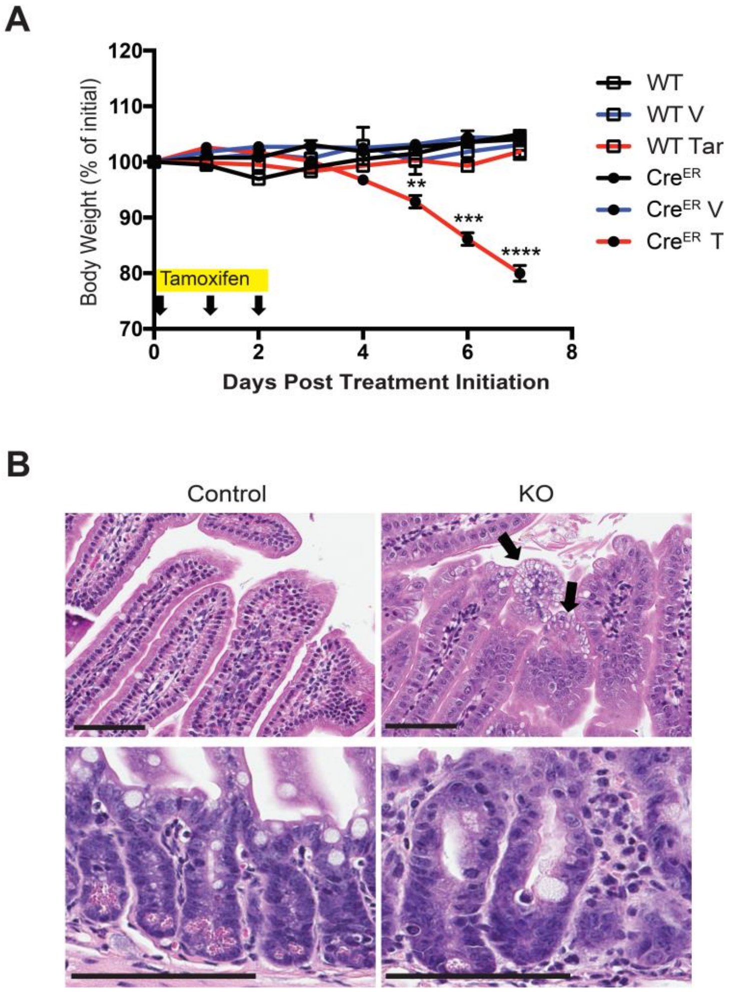

Figure S4. Clinical and histological features of tamoxifen-treated *ROSA-Cre^{ERT2+} EpCAM$^{fl/fl}$* mice mimic those of patients with CTE. (*A*) Weight loss accompanying acute deletion of EpCAM in tamoxifen-treated *ROSA-Cre^{ERT2+} EpCAM$^{fl/fl}$* mice. Tamoxifen (0.2 mg/g body weight) in vehicle (Tam), or vehicle (V) was administered to control or *ROSA-Cre^{ERT2+} EpCAM$^{fl/fl}$* mice for 3 d by gavage as indicated and serial body weights were determined (mean ± SEM). **$P < .01$, ***$P < .001$ and ****$P < .0001$ in comparisons between tamoxifen-treated WT and Cre-expressing mice using Student's t test (mean ± SEM). (*B*) Tufted enterocytes and elongated intestinal crypts demonstrated in H&E stained sections of EpCAM KO (tamoxifen-treated *ROSA26-Cre^{ERT2+} EpCAM$^{fl/fl}$*) jejunal tissue. Bars = 100 µ. Lower panels of (*B*) correspond to lower panels of Figure 5D. Aggregate data from seven independent experiments (*A*) and representative images from 1 of 3 independent experiments (*B*) are shown.

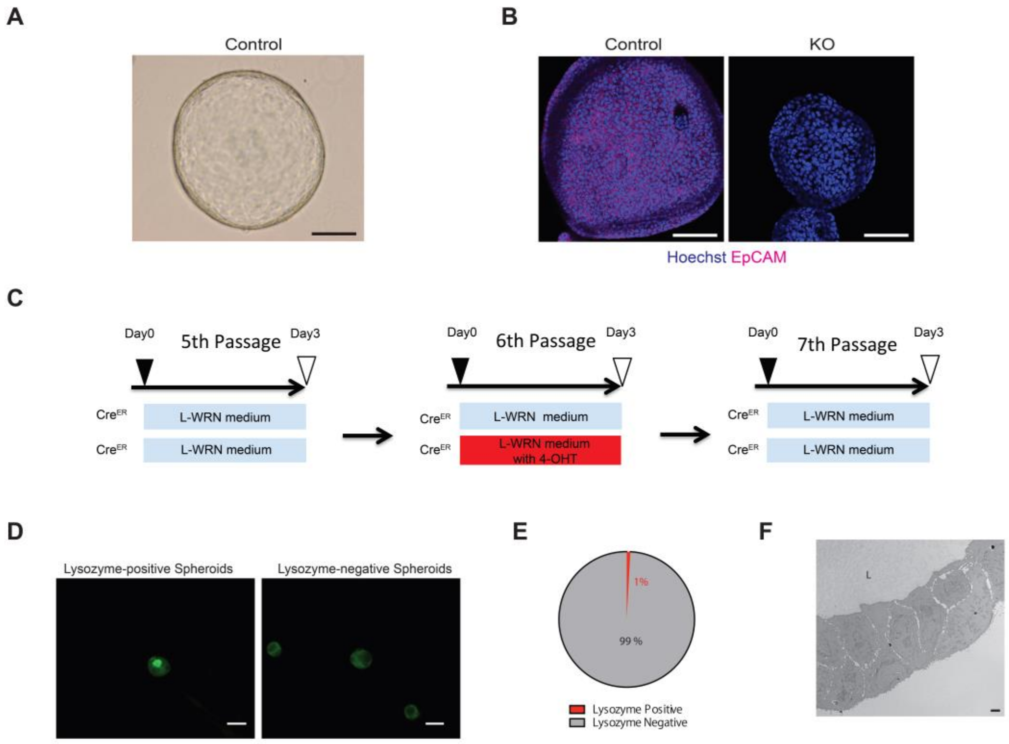

Figure S5. Establishment and characterization of EpCAM-expressing and EpCAM-deficient IEC spheroids. (*A*) Representative image of a stem cell-enriched spheroid on Day 3 of the 5th passage. Bar = 100μ. (*B*) EpCAM expression in IEC spheroids documented using confocal immunofluorescence microscopy (left). EpCAM expression is efficiently deleted in KO spheroids after 4-OHT treatment (right). Bar = 100 μ. EpCAM (pink); Hoechst 33342 counterstain (blue). (*C*) Schematic depicting strategy for studying EpCAM function in spheroids. Spheroids were treated with 4-OHT for 3 d during the 6th passage. 4-OHT-treated (KO) and untreated (Control) spheroids were then passaged. (*D*) Loss of Paneth cells from EpCAM-expressing IEC spheroids after serial passage. Representative immunofluorescence microscopic images of lysozyme$^+$ Paneth cell-containing (left panel) and Paneth cell-lacking (right panel) spheroids are shown. Bar = 100 μ. (*E*) Assessment of Paneth cell content in IEC spheroids as assessed by immunofluorescence microscopy (233 spheroids were counted (lysozyme$^+$ spheroids = 2, lysozyme$^-$ spheroids = 231). (*F*) Representative transmission electron micrographs of IEC spheroids and documenting morphology of epithelial cells and absence of Paneth cells. L = spheroid interior (lumen). Bars = 2 μ.

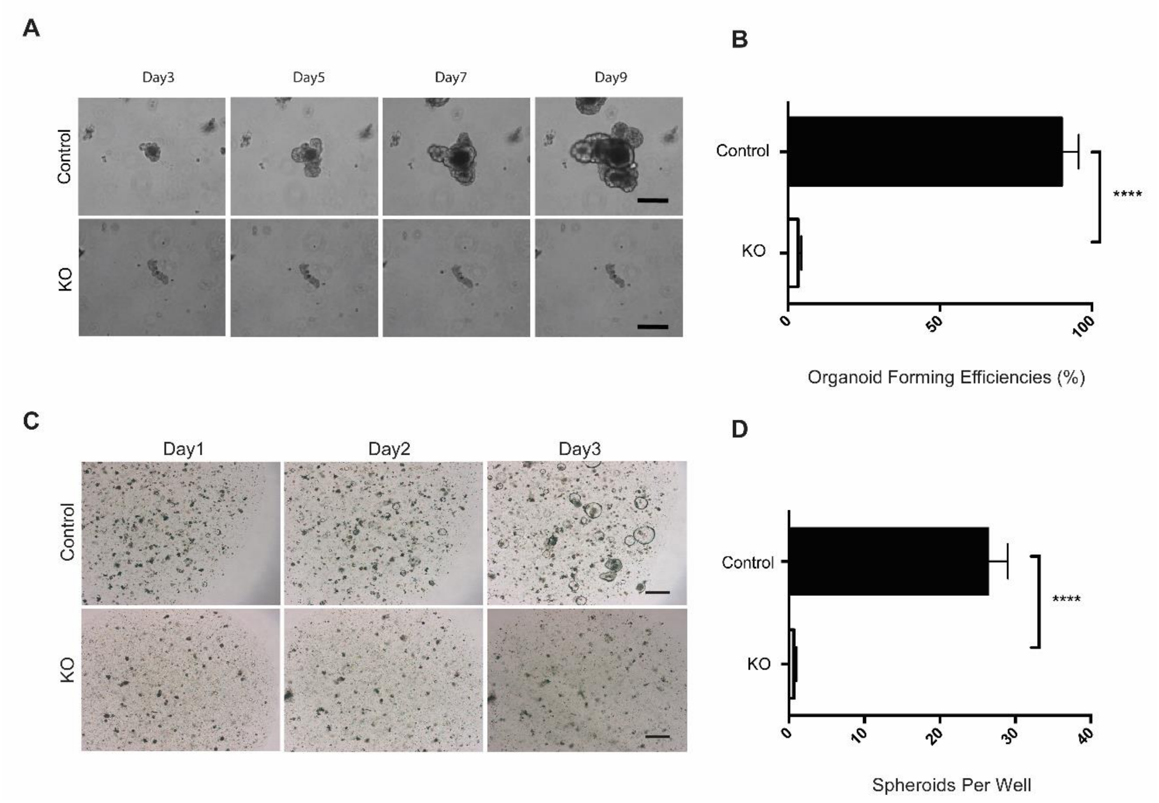

Figure S6. IEC organoids and spheroids cannot be efficiently generated from mice in which EpCAM has been acutely deleted. Adult *ROSA26-Cre*$^{ERT2+}$ *EpCAM*$^{fl/fl}$ mice were treated with tamoxifen or vehicle by gavage on Days 0-2 and jejunal tissue was obtained on Day 7. (*A*) Representative phase contrast photomicrographs of organoids generated from control and KO mice are depicted. Bars = 100 μ. (*B*) Quantitation of organoid forming efficiencies from crypts isolated from control and KO mice assessed on Day 9 after initiation of culture. **** $P < .0001$ via Student's t test. (*C*) Representative phase contrast photomicrographs of spheroids generated from control and KO mice are depicted. Bars = 100 μ. (*D*) Spheroid numbers per well from crypts isolated from control and KO mice assessed on Day 3 after initiation of culture. **** $P < .0001$ via Student's t test. Data depicted represent mean ± SEM in (*B*) and (*D*). Representative data from 1 of 3 independent experiments is shown in (*A*) and (C) and aggregate data from three independent experiments is shown in (*B*) and (*D*).

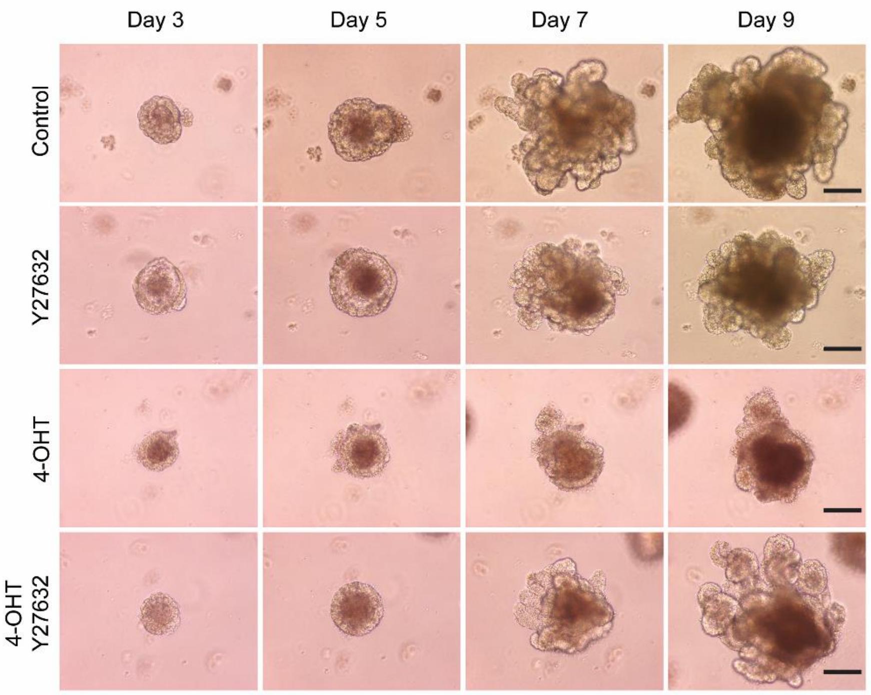

Figure S7. Effects of Y27632 on control and KO IEC organoids. Isolated crypts from *ROSA26-Cre*$^{ERT2+}$ *EpCAM*$^{fl/fl}$ mice were cultured as indicated and representative phase contrast photomicrographs were obtained at the indicated times. Bars = 100 μ. Representative data from 1 of 3 independent experiments are shown.

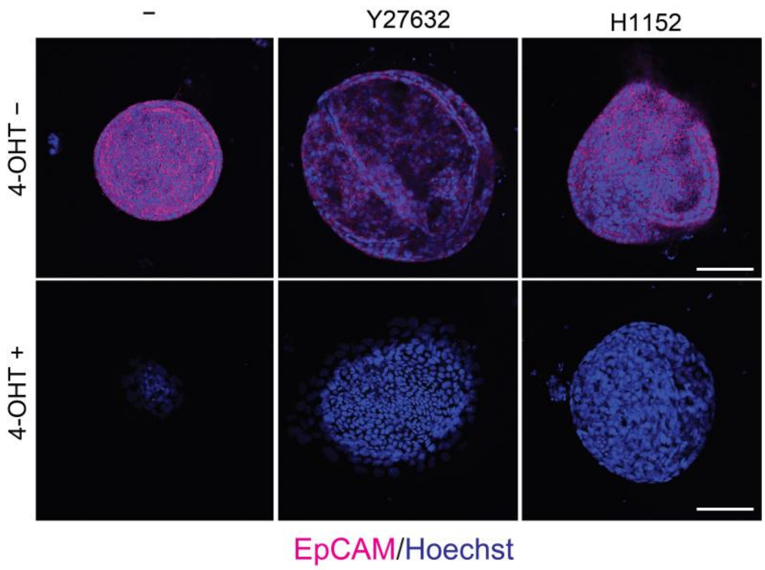

Figure S8. KO spheroids that are propagated in ROCK inhibitors do not express EpCAM. IEC spheroids from *ROSA26-Cre*$^{ERT2+}$ *EpCAM*$^{fl/fl}$ mice were treated with 4- OHT and ROCK inhibitors during their 6th passage and passed again in the presence and absence of ROCK inhibitors. Lack of EpCAM expression in KO spheroids was confirmed by confocal immunofluorescence microscopy. EpCAM (pink); nuclear counterstain Hoechst 33342 (blue). Bars = 100 μ. Representative data from 1 of 2 independent experiments are shown.

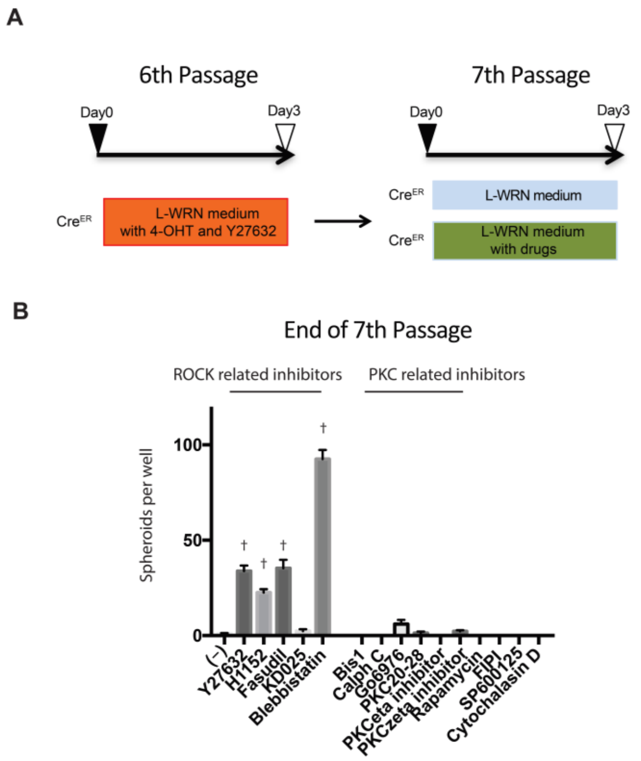

Figure S9. Effects of ROCK1 and myosin II inhibitors on EpCAM KO IEC spheroids are selective. (*A*) Schematic describing strategy for drug treatment and passaging of IEC spheroids. IEC spheroids from *ROSA26-Cre*$^{ERT2+}$ *EpCAM*$^{fl/fl}$ mice were treated with 4-OHT and Y27632 during their 6th passage and passed again in the presence and absence of the indicated inhibitors. Sixth passage KO spheroids were successfully propagated in the presence of the ROCK inhibitor Y27632. (*B*) Numbers of spheroids per well were counted on Day 3 of the 7th passage in the presence and absence of inhibitors. ROCK I inhibitors Y27632 (10 μM), H1152 (0.31 μM) and Fasudil (10 μM), as well as KD025 (a ROCK II inhibitor, 0.31 μM), blebbistatin (a myosin II inhibitor, 10 μM), Bis 1(a generic PKC inhibitor, 500 nM), Calph C (a PKCγ,δ inhibitor, 0.2 μM), Go6976 (a PKCα,β,γ inhibitor, 20 nM), PKC20-28 (a PKCα,β inhibitor, 20 μM), PKCeta inhibitor (25 ng/μl), PKCzeta inhibitor (10 μM), rapamycin (an mTOR inhibitor, 100 nM), FIPI (a phospholipase D inhibitor, 50 μM), SP600125 (a JNK inhibitor, 50 μM), Cytochalasin D (an actin polymerization inhibitor, 2 μM) were tested. † $P < .0001$ in comparisons between no drug and drugs using Student's t test (mean ± SEM). Aggregate data from three independent experiments are shown.

Supplementary Materials and Methods

Mice and genotyping

Mice were bred and housed in a specific pathogen–free environment. Procedures and study protocols were approved by the NCI animal care and use committee. Age-matched adult (8-12 week old) male or female mice were used in experiments. *ROSA26 Cre*$^{ERT2+}$ mice were purchased from the Jackson Laboratory (Bar Harbor, ME) (B6.129-*Gt(ROSA)26Sor*$^{tm1(cre/ERT2)Tyj}$/J) and *EpCAM*$^{fl/fl}$ mice were generated in our laboratory[19]. *ROSA26 Cre*$^{ERT2+/-}$ mice were crossed with *EpCAM*$^{fl/fl}$ mice to generate *ROSA26 Cre*$^{ERT2+}$ *EpCAM*$^{fl/fl}$ (Cre^{ER} mice) and *ROSA26 Cre*$^{ERT2-}$ *EpCAM*$^{fl/fl}$ (WT mice). Mice were tested for the presence of the floxed *EpCAM* allele and relevant transgene using tail-clip DNA isolated using a Qiagen DNeasy Blood and Tissue Kit (Qiagen, Valencia, CA). PCR-based detection of floxed *EpCAM* alleles was performed using the following primers: upstream loxP site, ULXP-gt-F (5′-gcccaattctagtggggagtgtcatg-3′), ULXP-gt-R (5′-gcctccaggcttttgtctcagatcag-3′); downstream loxP site, DLXP-gt-F (5′-gctatcttaccaacatttcatg-3′), DLXP-gt-R (5′-gaaattctctccagaccgctttg-3′). The genotyping method and primers for *ROSA26 Cre*^{ERT2} were as described by the Jackson Laboratory.

Antibodies

The following antibodies were used for immunofluorescence microscopy experiments and immune blotting studies: rabbit anti-human lysozyme (Dako, Carpinteria, CA, A0099), rabbit anti-mouse mucin2 (Santa Cruz Biotechnology, Santa Cruz, CA, sc-15334), goat anti-mouse Chr-A (Santa Cruz Biotechnology, sc-1488), goat anti-mouse villin (Santa Cruz Biotechnology, sc-7672), rabbit anti-mouse ZO-1 N-term (Invitrogen, Carlsbad, CA, 402300), rabbit anti-mouse claudin-1 (Invitrogen, 349100), rabbit anti-mouse claudin-7 (Invitrogen, 519000) , rabbit anti-mouse cleaved caspase-3 (Cell Signaling Technology, Beverly, MA, #9664), rabbit anti-mouse occludin (Invitrogen, 711500), rat anti-mouse E-Cadherin (eBioscience, San Diego, CA, 14-3249 or Takara, Tokyo, Japan, M108), rabbit anti-human (mouse) desmoglein-2 (Dsg2) (Abcam, Cambridge, MA, ab96761, ab150372), rabbit anti-mouse Na+/K+ ATPase alpha (Abcam, ab76020), rabbit anti-mouse phospho-histone H3, Ser10 (Cell Signaling Technology, #3377), Alexa 647-conjugated phalloidin (Cell Signaling Technology, #8940), Alexa 647-conjugated rat anti-mouse EpCAM (Biolegend, San Diego, CA, 118211), rat anti-mouse EpCAM (produced by hybridoma for G8.8), rabbit anti-mouse phospho-myosin light chain 2 (Ser19) (Cell Signaling Technology #3671), rabbit anti-mouse phospho-myosin light chain 2 (Thr18/Ser19) (Cell Signaling Technology #3674), mouse β-actin (Sigma-Aldrich, A1978). Secondary Abs were the following: Alexa 568-conjugated goat anti-rabbit IgG (Invitrogen, A-11077), Alexa 488-conjugated donkey anti-rabbit IgG (Invitrogen, A-21206), Alexa 488-conjugated donkey anti-rat IgG (Jax, 712-546-150), Alexa

488-conjugated goat anti-rabbit IgG (Invitrogen, A-11034), donkey HRP-conjugated anti-rabbit (Jackson ImmunoResearch Laboratories, West Grove, PA, 711-035-152), donkey HRP-conjugated anti-mouse (Jackson ImmunoResearch Laboratories, 715-036-150), goat HRP-conjugated anti-rabbit (Cell Signaling Technology, #7074).

IEC organoid generation and propagation

IEC organoids were generated using methodology developed by Clevers and coworkers [21-24]. Crypts were isolated from small intestines of WT and Cre^{ER} mice via treatment with 5 mM EDTA in PBS for 30 min at 4 °C with mechanical dissociation and subsequently cultured in growth factor-supplemented media as reported previously [21-24]. Briefly, isolated crypts were embedded in 50 μl of Matrigel (BD Bioscience, San Jose, CA) in 24 well plates, and 600 μl of advanced DMEM/F12 medium (Invitrogen) containing Penicillin-Streptomycin (Gibco, Grand Island, NY), Glutamax™ (Gibco), Epithelial Growth Factor (40 ng/ml, PeproTech, Rocky Hill, NJ), Noggin (100 ng/ml, Peprotech) and R-spondin-1 (500 ng/ml, R&D Systems, Minneapolis, MN) was added after Matrigel polymerization. Culture media was exchanged every two days.

Organoid forming efficiencies were determined by dividing the number of mature organoids per well on Day 9-11 by the mean number of organoids per well present on Day 1 and multiplying by 100. This approach was more reliable that attempting to enumerate the number of crypts put into cultures on Day 0. As indicated, IEC organoids were passed on Day 7-9 after initiation of primary cultures by splitting 1 well into 5 wells as previously described [21-24]. Briefly, Matrigel discs were broken up by pipetting in advanced DMEM/F12 medium (Invitrogen) containing Penicillin-Streptomycin and Glutamax™. Organoids were mechanically dissociated into crypt-like budding domains by vigorous trituration and incompletely disrupted aggregates were removed by filtration through 70 μ nylon mesh. Filtrate was suspended in fresh Matrigel and subcultures were initiated in growth factor-supplemented media. Organoid passage efficiencies represent the ratio of the absolute number of mature organoids in 5 wells on Day 9 after subculturing (splitting 1 well into 5 wells) and the average number of mature organoids in individual wells on Day 9 before subculturing times 100.

IEC spheroid generation and propagation

IEC spheroids were generated using methodology developed by Stappenbeck and coworkers [25]. Mouse jejunum was harvested, washed and villi were removed by scraping the mucosa with a glass slide as described and discarded. After mincing, residual tissue fragments were disassociated in 2 mg/ml Type 1 Collagenase (ThermoFisher Scientific, Waltham, MA) with vigorously pipetting before incubation for 5-10 min at 37° C. This process was repeated 3-5 times. Epithelial cell units (including crypts) were passed through 70 μ nylon mesh, washed and

cultured in 50% L-WRN conditioned medium for 2-3 days. 50% L-WRN conditioned medium was prepared using an L cell line (L-WRN cells, ATCC) engineered to secrete R-spondin3, Noggin and Wnt3a and primary culture medium (advanced DMEM/F12 supplemented with 20% FBS, L-glutamine, penicillin and streptomycin). Y27632 (10 μM) and SB431542 (10 μM), both from R&D Systems, were added to media during passages 1-5 only. Spheroids were passed after vigorous pipetting in 0.25% trypsin-EDTA (ThermoFisher Scientific) every 2-3 days. Because spheroid growth became reproducible after 4 passages, we used spheroids that had been passed 5-7 times in all experiments. Passage 5 spheroids were frozen and thawed for subsequent use as necessary for convenience and to improve experimental reproducibility [24].

Spheroid forming numbers were determined by counting numbers of spheroids per well on day 3 of the 6th passage in the presence and absence of Y27632 (10 μM), H1152 (0.31 μM) and 4-OHT (Sigma-Aldrich, St. Louis, MO, 1 μM) as indicated. Spheroids were passed 1:1 and passage numbers were determined by counting numbers of spheroids per well on day 3 of the 7th passage in the continued presence of inhibitors. Only spheroids with diameters ≥100 μ as determined by inverted phase light microscopy were counted. Numbers of spheroids observed in the presence of Y27632 and H1152 are expressed relative to numbers of control spheroids.

Conditional deletion of *EpCAM in vitro*

To activate *ROSA26 Cre*^{ERT2}, isolated crypts or spheroids were incubated with culture medium containing 1 μM of 4-OHT for 72 h. In preliminary experiments, we determined that 72 h is the minimum 4-OHT exposure time that resulted in complete disruption of *EpCAM* (data not shown).

Conditional deletion of *EpCAM in vivo*

To induce acute *EpCAM* deletion *in vivo*, adult *ROSA26 Cre*$^{ERT2+}$ *EpCAM*$^{fl/fl}$ mice were treated with tamoxifen (0.2 mg/g body weight, Sigma-Aldrich) daily for 3 days via gavage. Tamoxifen (33 mg/ml) was dissolved in Sunflower oil (Vehicle) prior to administration.

Routine and confocal immunofluorescence microscopy

Organoids and spheroids cultured in 24 well plates were fixed *in situ* with 4% paraformaldehyde in PBS for 20 min at room temperature (RT) prior to isolation from Matrigel discs via gentle pipetting. Fixed organoids and spheroids were subsequently permeabilized with 0.1% Triton X-100 for 15 min at RT followed by blocking with 1% bovine serum albumin before overnight incubation with primary antibodies at 4° C with gentle rotation. In spheroid experiments for immunofluorescence staining, Nunc Lab-Tek II Chamber Slide System 8-well plates (ThermoFisher Scientific) were used instead of 24 well plates.

Phase contrast images of organoids and spheroids were obtained with an inverted microscope (Nikon Eclipse TE2000). Confocal immunofluorescence images were captured with a laser-scanning confocal microscope (LSM710 or LSM780; Carl Zeiss Micro imaging) equipped with a 63x 1.40 oil plan-apochromat objective using 0.4 μ optical slices, and data were processed using an LSM Image Browser (Zeiss) and ZEN (Zeiss).

To objectively assess phosphorylation of myosin light chain, images were acquired using a Zeiss LSM780 confocal microscope equipped with a 20x plan-apochromat (N.A. 0.8) objective lens. Confocal images were collected with 0.42μ X-Y pixel size and an optical slice thickness of 2.0 μ. The resultant images were analyzed using the Zeiss Zen (v.2) image processing and analysis software. Mean intensities of the background staining were measured from the isotype negative immunolabeling control and this background was subtracted from the remaining images. After background subtraction, an image mask was applied to the Alexa fluor 488 (green) channel to mark the regions of interest for measuring the mean pixel intensity in all three fluorescence channels. The threshold value used to generate the image mask was applied to all images in the dataset.

2.6. RNA isolation and qPCR

Wells were rinsed with ice-cold PBS and organoids were harvested by gentle trituration. Total RNA was isolated from organoids using Trizol reagent (Invitrogen) and RNAeasy mini kits (Qiagen). cDNA was prepared using AffinityScript Multiple Temperature cDNA Synthesis kits according to the manufacturer's (Stratagene) protocol. Quantitative real-time PCR (qPCR) was performed using a Bio-Rad CFX96 Real-time thermos system (Bio-Rad, Hercules, CA) and PowerSYBR Green PCR master mix (PE Biosystems, Foster City, CA) with Bio-Rad provided PrimerPCR SYBR Green Assay primers specific for mouse *EpCAM*. Individual samples were normalized using β-actin mRNA-derived signals. EpCAM mRNA levels are expressed relative to β-actin mRNA levels as calculated by the 2-ΔΔCT method where ΔCT=CT(EpCAM)-CT(β-actin)

Western blotting

Wells were rinsed with ice-cold PBS containing phosphatase inhibitors (1 mM of sodium fluoride and 1mM sodium orthovanadate) and organoids and spheroids were subsequently harvested with ice-cold PBS/phosphatase inhibitors by gentle pipetting. Organoids and spheroids were then sedimented at 150 G for 3 min at 4°C, washed with ice-cold PBS with phosphatase inhibitors, suspended in cold lysis buffer (1% Triton X-100, 0.1% SDS, 150 mM NaCl, 50mM Tris pH 8.0, 1 mM EDTA, 0.25 % sodium deoxycholate, 10mM sodium fluoride, protease inhibitor cocktail (cOmplete ULTRA Tablets, Mini, EDTA-free, EASYpack, Roche, Switzerland), and phosphatase inhibitor cocktail (PhosSTOP, Roche) and sonicated using a Bioruptor

(Diagenode) on ice. For *in vivo* experiments, small intestine was homogenized directly into lysis buffer. Protein concentrations were determined using a BCA protein assay (Invitrogen). Equal amounts of protein were incubated at 70° C for 10 min in Nupage LDS sample buffer (Invitrogen) and Nupage sample reducing reagent (Invitrogen) and resolved by Nupage 4–12% or 12% Bis-Tris polyacrylamide gel electrophoresis (Invitrogen) prior to blotting onto polyvinylidene difluoride membranes using an iBlot dry blotting system (Invitrogen). Membranes were blocked with 2%BSA in Tris Buffered Saline with 0.1% Tween20 for 1 h and then incubated with primary Abs in 1%BSA/TBST overnight at 4°C with gentle shaking. After washing, membranes were incubated with secondary Abs in 1%BSA/TBST for 1 h at RT with gentle shaking. After additional washing, membranes were exposed to SuperSignal West Dura or Pico Chemiluminescent Substrate (Thermo Scientific, #34075 or #34077) and labeled proteins were detected with photographic film.

EdU incorporation (cell proliferation) assay

Proliferating IEC in organoids were visualized using a Click-iT EdU imaging kit (Invitrogen) and immunofluorescence microscopy in accordance with the manufacturer's instructions.

TUNEL (apoptosis) assay

Apoptotic cells were visualized in IEC organoids using EdUTP and a Click-iT TUNEL imaging kit (Invitrogen) in conjunction with immunofluorescence microscopy.

Transmission electron microscopy

Organoids and spheroids in Matrigel were fixed with sodium cacodylate buffer (0.1 M, pH 7.4) containing 2% (v/v) EM grade glutaraldehyde (Tousimis, Rockville, MD) and 4% (v/v) formaldehyde for 2 h at RT followed by extensive washing in same buffer. Post-fixation was accomplished in 1% (v/v) osmium tetroxide (Electron Microscopy Sciences) in sodium cacodylate buffer for 1 h at RT. Small pieces of Matrigel containing organoids or spheroids were transferred into glass scintillation vials for dehydration. Organoids or spheroids were dehydrated in an ethanol series (35%, 50%, 75%, 95%, and 100%) followed by 100% propylene oxide. Infiltration was carried out in an equal mixture of 100% propylene oxide and epoxy resin (Electron Microscopy Sciences) overnight. Organoids or spheroids were embedded in fresh epoxy resin the next day and cured in a 55° C oven for 48 h. The cured epoxy block was thin-sectioned (60 to 70 nm), mounted on 200 copper mesh grids, and stained with uranyl acetate and lead citrate. Grids were examined in an electron microscope (H7600, Hitachi) operated at 80kv and digital images were captured with a CCD camera (AMT).

Small molecule penetration assay

To assess epithelial integrity, 1 mg/ml EZ-link sulfo-NHS-LC-biotin (ThermoFisher Scientific) was added to organoid culture medium. Organoids were cultured for an additional 4 h at 37 °C, and then fixed and permeabilized as described above. Biotinylated proteins were detected with Alexa fluor 568-streptavidin via fluorescence microscopy.

***In vitro* rescue experiments with selective drugs**

The following inhibitors and growth factors were added to culture medium at the indicated concentrations: 10 μM trans-4-[(1*R*)-1-aminoethyl]-*N*-4-pyridinylcyclohexanecarboxamide dihydrochloride (Y27632, Sigma-Aldrich), 0.31 μM (S)-(+)-2-Methyl-1-[(4-methyl-5-iscquinolinyl) sulfonyl]-hexahydro-1*H*-1,4-diazepine dihydrochloride (H1152, TOCRIS, Ellisville, MO), 10 μM 1-Phenyl-1,2,3,4-tetrahydro-4-hydroxypyrrolo[2.3-b]-7-methylquinolin-4-one (Blebbistatin, Sigma-Aldrich), 100 ng/ml of Wnt-3a (R&D, 1324-WN), 0.31 μM of 2-[3-[4-(1H-indazol-5-ylamino)-2-quinazolinyl]phenoxy]-N-(1-methylethyl)- acetamide (KD025, Sellckchem, No.S7936), 10 μM of Bisindolylmaleinmide-I (generic PKC inhibitor) (EMD Millipore, 203290, Billerica, MA), 0.31 μM of (1R)-2-[12-[(2R)-2-(Benzoyloxy)propyl]-3,10-dihydro-4,9-dihydroxy-2,6,7,11-tetramethoxy-3,10-dioxo-1-perylenyl]-1-methylethylcarbonic acid 4-hydroxyphenyl ester (Calphostin C, PKCγ,δ inhibitor) (EMD Millipore, 208725), 10 μM of 5,6,7,13-Tetrahydro-13-methyl-5-oxo-12*H*-indolo[2,3-*a*]pyrrolo[3,4-*c*]carbazole-12-propanenitrile (Go6976, PKCα,β,γ inhibitor) (EMD Millipore, 365250), 0.31 μM of PKC20-28 (PKCα,β inhibitor) (EMD Millipore, 476480), 10 μM of PKCeta inhibitor (Santa Cruz, sc-3096), 10 μM of PKCzeta pseudosubstrate (TOCRIS, No1791), 0.31 μM of (3S,6R,7E,9R,10R,12R,14S,15E,17E,19E,21S,23S,26R,27R,34aS)-9,10,12,13,14,21,22,23,24,25,26,27,32,33,34,34a-Hexadecahydro-9,27-dihydroxy-3-[(1R)-2-[(1S,3R,4R)-4-hydroxy-3-methoxycyclohexyl]-1-methylethyl]-10,21-dimethoxy-6,8,12,14,20,26-hexamethyl-23,27-ep (Rapamycin, mTOR inhibitor) (Sigma-Aldrich, R0395), 10 μM of 5-fluoro-N-[2-[4-(2-oxo-3H-benzimidazol-1-yl)piperidin-1-yl]ethyl]-1H-indole-2-carboxamide; 5-Fluoro-N-(2-(4-(2-Oxo-2,3-dihydro-1H-benzo[d]imidazol-1-yl)piperidin-1-yl)ethyl)-1H-indole-2-carboxamide (FIPI, phospholipase D inhibitor) (Sigma-Aldrich, F5807), 0.31 μM of 1,9-Pyrazoloanthrone (SP600125, JNK inhibitor) (Sigma-Aldrich, S5567), 10 μM of (7S,13E,16S,18R,19E,21R)-21-(Acetyloxy)- 7,18-dihydroxy-16,18-dimethyl-10-phenyl[11]cytochalasa-6(12),13,19-triene-1,17-dione (Cytochalasin D, actin polymerization inhibitor) (Sigma-Aldrich, C8273).

In experiments with organoids, crypts isolated from Cre^{ER} mice were cultured with normal medium (1st group), culture medium supplemented with inhibitors (2nd group), culture

medium containing 1 μM of 4-OHT (3rd group) and medium supplemented with inhibitors and 1 μM of 4-OHT (4th group) for 3 days after crypt isolation. Organoids in groups 1 and 3 were then cultured with normal culture medium for an additional 6 d. Orgnaoids in groups 2 and 4 were then cultured with the medium supplemented with inhibitors for additional 6 h. In experiments testing passage efficiencies, groups 2 and 4 continued to be cultured with the inhibitor-supplemented medium. In experiments with spheroids, 6th passage spheroids were treated with inhibitors and 1 μM of 4-OHT for 3 d as in the case of organoids. During the 7th passage, groups 2 and 4 continued to be cultured with the medium supplemented with inhibitors.

RNA in situ hybridization (FISH)

Duplex fluorescence RNA in situ hybridization was performed. Briefly, organoids were cultured for 9 d in the presence and absence of 4-OHT and Y27632 and then liberated from Matrigel by pipetting with cold 2 mM EDTA in PBS. Organoids from multiple wells were pooled and sedimented at 200g for 3 min. Pellets were washed with cold PBS, re-suspended organoids were transferred into a 1.5ml centrifuge tube and centrifuged at 8000 rpm for 30 sec. Eight μl of thrombin (1 unit/μl, Sigma # T-7009) and 5 μl of fibrinogen (10 mg/ml, Sigma #F-3879) were added. Clotted organoid pellets were fixed in fresh 4% paraformaldehyde for 24 h at RT and subsequently transferred to specialized screen processing cassettes (Thermo Scientific Tissue-Loc HistoScreen). As indicated, segments of mouse intestine were fixed in fresh 4% paraformaldehyde for 24 h at RT. Representative cross sections approximately 3 mm in length were taken from each intestinal region, individually placed into tissue processing cassettes as described. Cassettes were stored in 70% EtOH at RT prior to paraffin embedding. Tissue processing and paraffin infiltration was accomplished using an automated vacuum infiltration tissue processor (Sakura Tissue Tek VIP) with processing from 70% EtOH through graded ethanol, Xylene, and paraffin infiltration. Processing protocol times were 20 mins per reagent.

Paraffin embedded samples were serially sectioned at 5 μ using a rotary microtome (Leica Microsystems). Sections were mounted onto positively charged glass microscope slides and slides were dried at 60°C for 1 h, dewaxed through a series of a xylenes (2 x 5 min) and alcohols (2 x 3 min), and dried overnight at RT. Access to cellular RNA was made available by boiling slides for 30 min in Pretreatment 2 followed by a 20 min incubation at 40°C of a 1:5 dilution or 1:10 dilution, for intestinal tissue and organoid pellets respectively, of Protease III. Approximately 200 μl of either 3-plex negative control probe, Defa1-C1, Olfm4-C2, or Defa-C1/Olfm4 duplex probes were hybridized for 2 h at 40°C followed by washing twice with 0.5X Wash Buffer for 2 min. Signal was amplified by incubating slides at 40°C with Fluorescent Amplification 1 solution for 30 min, Amplification 2 solution for 15 min, Amplification 3 solution for 30 min, and Amplification 4 Alt A (Probe C1-Alexa 488 and Probe C2-Atto 550) solution for 15

min with two 2 min washes in 0.5X Wash buffer after every incubation. Slides were counter stained with DAPI and cover slipped with ProLong® Gold Antifade Reagent (Life Technologies). Slides were dried at RT overnight and scanned at 20X magnification using an Aperio ScanScope FL with an Ex:377/$\pm$50, Em:447/$\pm$60 filter for DAPI counterstain, an Ex:482/$\pm$35, Em:536/$\pm$40 filter for Alexa 488, and an Ex:543/$\pm$22, Em:593/$\pm$40 filter for Atto 550 visualization.

Statistical analysis

Statistical analysis was performed using Mann–Whitney *U* test or one-way ANOVA test via Prism5 or 7 software (GraphPad). p-values $\leq$0.05 were regarded to be significant.
